# Digging up the roots of an insular hotspot of genetic diversity: decoupled mito-nuclear histories in the evolution of the Corsican-Sardinian endemic lizard *Podarcis tiliguerta*

**DOI:** 10.1186/s12862-017-0899-x

**Published:** 2017-03-02

**Authors:** Daniele Salvi, Catarina Pinho, D. James Harris

**Affiliations:** 1 Department of Health, Life and Environmental Sciences, University of L’Aquila, 67100 Coppito, L’Aquila, Italy; 2 CIBIO-InBIO, Centro de Investigação em Biodiversidade e Recursos Genéticos, Campus Agrário de Vairão, 4485-661 Vairão, Portugal

**Keywords:** Cyto-nuclear discordance, Evolutionary history, Genetic diversity, Local adaptation, Mediterranean island, Phylogeography

## Abstract

**Background:**

Mediterranean islands host a disproportionately high level of biodiversity and endemisms. Growing phylogeographic evidence on island endemics has unveiled unexpectedly complex patterns of intra-island diversification, which originated at diverse spatial and temporal scales. We investigated multilocus genetic variation of the Corsican-Sardinian endemic lizard *Podarcis tiliguerta* with the aim of shedding more light on the evolutionary processes underlying the origin of Mediterranean island biodiversity.

**Results:**

We analysed DNA sequences of mitochondrial (*12S* and *nd4*) and nuclear (*acm4* and *mc1r*) gene fragments in 174 individuals of *P. tiliguerta* from 81 localities across the full range of the species in a geographic and genealogical framework. We found surprisingly high genetic diversity both at mitochondrial and nuclear loci. Seventeen reciprocally monophyletic allopatric mitochondrial haplogroups were sharply divided into four main mitochondrial lineages (two in Corsica and two in Sardinia) of Miocene origin. In contrast, shallow divergence and shared diversity within and between islands was observed at the nuclear loci. We evaluated alternative biogeographic and evolutionary scenarios to explain such profound discordance in spatial and phylogenetic patterning between mitochondrial and nuclear genomes. While neutral models provided unparsimonious explanations for the observed pattern, the hypothesis of environmental selection driving mitochondrial divergence in the presence of nuclear gene flow is favoured.

**Conclusions:**

Our study on the genetic variation of *P. tiliguerta* reveals surprising levels of diversity underlining a complex phylogeographic pattern with a striking example of mito-nuclear discordance. These findings have profound implications, not only for the taxonomy and conservation of *P. tiliguerta*. Growing evidence on deep mitochondrial breaks in absence of geographic barriers and of climatic factors associated to genetic variation of Corsican-Sardinian endemics warrants additional investigation on the potential role of environmental selection driving the evolution of diversity hotspots within Mediterranean islands.

**Electronic supplementary material:**

The online version of this article (doi:10.1186/s12862-017-0899-x) contains supplementary material, which is available to authorized users.

## Background

The Mediterranean Basin has long been recognised as one of the richest global biodiversity hotspots [1]. A large fraction of such diversity, and particularly endemic diversity, is hosted within the over 5,000 islands scattered throughout the Mediterranean Sea [2]. The level of diversity and endemism is especially high in large continental islands such as the Tyrrhenian islands Corsica and Sardinia, which are considered a regional biodiversity hotspot within the Mediterranean area [2–4].

These islands offer an ideal setting for investigating the evolutionary processes behind the origin and the structure of current patterns of insular biodiversity hotspots because: (i) they have a complex topography with a diversity of landscapes and microclimatic regions, spanning from Mediterranean to alpine climates [5, 6], which combined with the imprints of Plio-Pleistocene climatic oscillations [7–10] contribute to the diversification and persistence of old lineages [11–13]; (ii) the palaeogeographical evolution of the Corsican-Sardinian system within the Western Mediterranean is well established [14–18], thus providing a useful framework for biogeographic and molecular inferences [19]; and (iii) emerging phylogeographic and phylogenetic data on many endemics allow conclusions to be drawn within a comparative framework [see e.g. [19, 20].

In the last decade, Corsican-Sardinian species have been the subject of intensive phylogeographic surveys, especially regarding amphibians and reptiles [12, 13, 20–34], which have revealed how an essential component of the Tyrrhenian biodiversity hotspot is represented by the genetic variation held within and among populations of these endemic species. A significant realization of these studies is that the current patterns of genetic structure and diversity of these endemic species have been historically shaped by an unexpectedly diverse array of evolutionary and demographic processes acting across unrelated spatial and temporal scales [20]. In fact, essentially each phylogeographical reconstruction carried out so far on Corsican-Sardinian species suggested an idiosyncratic scenario for the evolution of the current geographical patterns of intraspecific genetic diversity [12, 19, 20, 30, 31]. This suggests that we are still far from either an exhaustive inventory or a deep understanding of the evolutionary processes underlying the origins and diversity of this biodiversity hotspot.

In regard to this, the Tyrrhenian wall lizard *Podarcis tiliguerta* offers an intriguing case study as previous genetic assessments uncovered extraordinarily high level of diversity with contrasting patterns between different genetic markers [21, 24]. This species is common and locally abundant across a variety of shrubby and open habitats [35] from the sea level (including tiny islets) up to 1800 m asl in the mountain regions [36]. Based on the current continuous distribution of *P. tiliguerta* within both Corsica and Sardinia [37, 38] and associated with the fact that these two islands were largely and persistently connected into a single landmass during the Pleistocene glaciations [18, 39], we may have expected low genetic differentiation between populations with most of the genetic diversity shared within and between the main islands. In contrast, preliminary mitochondrial datasets [24, 40, 41], based on a few individuals and short gene fragments, showed three highly divergent lineages, one in Corsica and two in Sardinia, with genetic distances between them exceeding those typically found between reptile species [24, 42]. On the other hand, allozyme data from 15 variable loci suggested a lower genetic distance between Corsican and Sardinian populations (with no alternatively fixed alleles) with a latitudinal clinal variation in allele frequencies at some loci and an overall isolation-by distance pattern suggesting reduced gene-flow between populations [21].

Therefore, while all previous studies found extraordinarily high genetic diversity and substantial differentiation between populations, mitochondrial data indicate that *P. tiliguerta* may be a species complex [24] whereas nuclear data depict *P. tiliguerta* as a single species geographically structured in local populations [21], suggesting a possible mito-nuclear discordance. Comparing the mitochondrial and nuclear patterns of diversity and levels of divergence found within *P. tiliguerta* by previous studies, and identifying the evolutionary processes underlying their formation, is difficult. Allozyme analyses lack a genealogical framework to understand the evolutionary relationships between alleles, and genetic diversity and divergence between populations may be underestimated due to the occurrence of iso-electrophoretic alleles (distinct alleles with equal electrophoretic mobility). On the other hand, mitochondrial assessments only account for a single genealogical realization and previous studies have likely underestimated the genetic diversity of *P. tiliguerta* due to the short gene fragment used, limited geographic sampling, and the low number of individuals analysed, as supported by the finding that new haplotypes and lineages were sampled as a few more individuals were added in each study [40, 41].

In this study, we investigated the phylogeographic structure and evolutionary history of *P. tiliguerta* based on mitochondrial and nuclear genealogies sampled across the entire geographic range of the species. We found an extraordinarily high level of genetic diversity which has deep roots in the mitochondrial genome whereas it shows minimal phylogenetic and geographic structure in the nuclear genome. We evaluated the role of biogeographic and evolutionary processes at different temporal and spatial scales to explain the origin of the striking pattern of genetic richness and mito-nuclear discordance found within *P. tiliguerta* and we discussed possible generalizations within the Tyrrhenian biodiversity hotspot.

## Methods

### Genetic data collection

**Fig. 1:**
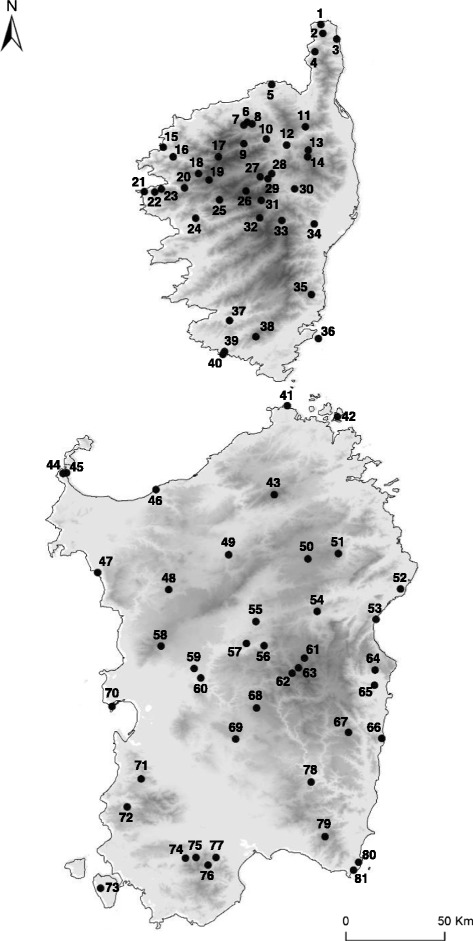
Map of the study area showing sampling locations of the Tyrrhenian wall lizard, *Podarcis tiliguerta*. Detailed information on sampling localities and specimens is found in Table 1

We sampled 174 individuals of *P. tiliguerta* from 81 localities across the entire species range (Fig. 1). Sampling design was informed by genetic analyses on a preliminary set of samples and refined during a four-year collection period (2009–2012). This was necessary to cover the high level of diversity found in *P. tiliguerta*. Individuals from additional 10 *Podarcis* species from Portugal, Spain, Italy, Malta, Slovenia, and Greece were collected and used in phylogenetic analyses together with sequences obtained from GenBank. Detailed information regarding individual and locality codes, geographic coordinates of sampling locality and GenBank accession numbers for all sequences used in this study is reported in Table 1. The source of data retrieved from GenBank is reported in Tables 2 and 3.

**Table 1:** Geographical location and codes for the studied populations and individuals of *Podarcis tiliguerta*. Genbank accession numbers of the sequences are provided for each gene

Locality Code	Specimen Code	Locality coordinates	Genbank accession numbers			
			*12S*	*nd4*	*mc1r*	*acm4*
1	C2.2	43.00 N 9.39 E	KY562009	KY562416	-	-
2	005	42.96 N 9.40 E	KY562010	KY562417	KY562320	-
3	C1.1	42.93 N 9.46 E	KY562011	KY562418	-	-
3	C1.2		KY562012	KY562419	-	-
4	C3.1	42.88 N 9.36 E	KY562013	KY562420	-	-
4	C3.2		KY562014	KY562421	-	-
5	C4.1	42.73 N 9.16 E	KY562015	KY562422	-	-
6	0018	42.55 N 9.05 E	KY562016	KY562423	-	-
7	0019	42.54 N 9.04 E	KY562017	KY562424	KY562412	-
8	394	42.55 N 9.07 E	KY562018	KY562425	KY562414	KY562281
9	383	42.46 N 9.04 E	KY562019	KY562426	KY562415	KY562282
10	380a	42.48 N 9.14 E	KY562020	KY562427	KY562299	KY562175
10	380b		KY562021	KY562428	KY562300	KY562176
10	380c		KY562022	KY562429	KY562301	KY562177
10	380d		KY562023	KY562430	KY562302	KY562178
11	0026	42.53 N 9.32 E	KY562024	KY562431	KY562303	-
12	0069a	42.45 N 9.23 E	KY562025	KY562432	-	-
12	0069b		KY562026	KY562433	-	-
13	395a	42.43 N 9.33 E	KY562027	KY562434	KY562304	KY562179
13	395b		KY562028	KY562435	KY562305	KY562180
14	0068a	42.40 N 9.33 E	KY562029	KY562436	-	-
14	0068b		KY562030	KY562437	-	-
15	C5.1	42.44 N 8.67 E	KY562031	KY562438	-	-
16	0081a	42.40 N 8.71 E	KY562032	KY562439	-	-
16	0081b		KY562033	KY562440	-	-
16	0081c		KY562034	KY562441	-	-
17	0060	42.40 N 8.92 E	KY562035	KY562442	-	-
17	388a		KY562036	KY562443	KY562291-2	KY562181
17	388b		KY562037	KY562444	KY562306	KY562182
17	388c		KY562038	KY562445	KY562307	-
18	0083a	42.32 N 8.83 E	KY562039	KY562446	KY562308	-
18	0083b		KY562040	KY562447	KY562309	-
18	0083c		KY562041	KY562448	-	-
19	387	42.29 N 8.88 E	KY562042	KY562449	KY562310	KY562183
20	0080a	42.26 N 8.77 E	KY562043	KY562450	KY562311	-
20	0080b		KY562044	KY562451	-	-
20	0080c		KY562045	KY562452	-	-
21	0075	42.24 N 8.58 E	KY562046	KY562453	-	-
21	S5A		KY562047	KY562454	KY562312	KY562184
21	S5B		KY562048	KY562455	KY562313	KY562185
22	C6.1	42.24 N 8.63 E	KY562049	KY562456	-	-
23	0076	42.25 N 8.66 E	KY562050	KY562457	-	-
24	0037	42.12 N 8.82 E	KY562051	KY562458	-	-
25	0072a	42.20 N 8.93 E	KY562052	KY562459	KY562314	-
25	0072b		KY562053	KY562460	-	-
25	0072c		KY562054	KY562461	-	-
26	RE1	42.24 N 9.05 E	KY562055	KY562462	KY562315	KY562186
26	RE2		KY562056	KY562463	KY562316	KY562187
26	RE3		KY562057	KY562464	KY562317	KY562188
26	RE4		KY562058	KY562465	KY562318	KY562189
27	0066a	42.31 N 9.11 E	KY562059	KY562466	-	-
27	0066b		KY562060	KY562467	KY562319	-
28	C7.1	42.32 N 9.16 E	KY562061	KY562468	-	-
29	0070	42.30 N 9.15 E	KY562062	KY562469	-	-
30	C8.1	42.25 N 9.27 E	KY562063	KY562470	-	-
31	0057a	42.20 N 9.12 E	KY562064	KY562471	KY562321	-
31	0057b		KY562065	KY562472	KY562322	-
32	0054	42.12 N 9.11 E	KY562066	KY562473	KY562323	-
32	377a		KY562067	KY562474	KY562324	KY562190
32	377b		KY562068	KY562475	KY562325	KY562191
33	373a	42.11 N 9.21 E	KY562069	KY562476	-	KY562192
33	373b		KY562070	KY562477	KY562326	-
33	373c		-	-	KY562327	KY562193
34	372	42.09 N 9.36 E	KY562071	KY562478	KY562328	KY562194
35	C9.1	41.77 N 9.35 E	KY562072	KY562479	-	-
36	CTg1	41.57 N 9.38 E	EF165024	KY562480	-	-
36	CTg2		EF165024	KY562481	-	-
36	CTg3		EF165024	KY562482	-	-
36	CTg4		EF165024	KY562483	-	-
37	369	41.65 N 8.97 E	KY562073	KY562484	KY562329	KY562195
38	401	41.58 N 9.09 E	KY562074	KY562485	KY562293-4	KY562200
38	367a		KY562075	KY562486	KY562330	KY562196
38	367b		KY562076	KY562487	KY562331	KY562197
38	367c		KY562077	KY562488	-	KY562198
38	367d		KY562078	KY562489	KY562332	KY562199
39	368a	41.51 N 8.95 E	KY562079	KY562490	KY562333	KY562201
39	368b		KY562080	KY562491	KY562334	KY562202
40	PEC1	41.49 N 8.94 E	KY562081	KY562492	KY562335	-
40	PEC2		KY562082	KY562493	KY562336	KY562203
40	PEC3		KY562083	KY562494	KY562337	KY562204
40	PEC4		KY562084	KY562495	KY562338	KY562205
40	PEC5		KY562085	KY562496	KY562339	-
41	PF1	41.26 N 9.24 E	KY562170	-	-	-
41	PF2		KY562086	KY562497	KY562340	KY562206
41	PF3		KY562087	KY562498	KY562341	KY562207
42	PET1	41.21 N 9.46 E	KY562088	KY562499	KY562342	KY562208
42	PET2		KY562089	KY562500	KY562343	KY562209
42	PET3	41.21 N 9.46 E	KY562090	KY562501	KY562344	KY562210
43	LIM1	40.85 N 9.17 E	KY562091	KY562502	KY562289-90	KY562211
43	LIM3		KY562092	KY562503	KY562345	KY562212
43	LIM4		KY562093	KY562504	KY562346	KY562213
43	LIM5		KY562094	KY562505	-	KY562214
44	db15006	40.95 N 8.21 E	KY562171	-	KY562347	KY562215
44	db15007		KY562172	-	KY562348	KY562216
45	STI	40.95 N 8.23 E	KY562173	-	KY562297-8	KY562217
46	db15424	40.88 N 8.64 E	KY562174	-	KY562349	KY562218
47	db1712	40.50 N 8.37 E	KY562095	KY562506	KY562350	KY562219
48	db15056	40.42 N 8.69 E	KY562154	-	KY562351	KY562220
49	db1708	40.58 N 8.97 E	KY562155	-	KY562352	-
50	db1706	40.56 N 9.33 E	KY562156	-	KY562353	-
50	db1707		KY562096	KY562507	KY562354	KY562221
51	db1704	40.58 N 9.47 E	KY562097	KY562508	KY562355	KY562222
51	db1705		KY562098	KY562509	KY562356	-
52	db1699	40.42 N 9.75 E	KY562099	KY562510	KY562357	KY562223
53	db1697	40.28 N 9.64 E	KY562100	KY562511	KY562358	KY562224
53	db1698		KY562101	KY562512	KY562359	KY562225
54	186a	40.32 N 9.37 E	KY562102	KY562513	KY562360	KY562226
54	186b		KY562157	-	KY562361	KY562227
54	186c		KY562103	KY562514	KY562362	-
54	db1693		KY562104	KY562515	KY562283-4	KY562228
54	db1694		KY562105	KY562516	KY562363	KY562229
55	db15259	40.27 N 9.09 E	KY562158	-	KY562364	KY562230
56	db15249	40.16 N 9.13 E	KY562159	-	KY562365	KY562231
57	db15255	40.17 N 9.05 E	KY562160	-	KY562366	KY562232
58	db15072	40.16 N 8.66 E	KY562161	-	KY562367	KY562233
58	db15084		KY562162	-	KY562368	KY562234
59	db15064	40.06 N 8.81 E	KY562163	-	KY562369	-
59	db15100		KY562164	-	KY562370	KY562235
60	db15053	40.02 N 8.84 E	KY562165	-	KY562371	KY562236
60	db15075		KY562166	-	KY562372	KY562237
61	db15143	40.11 N 9.31 E	KY562167	-	KY562373	KY562238
61	db15239		KY562168	-	KY562374	KY562239
62	328a	40.04 N 9.26 E	KY562106	KY562517	-	KY562240
62	328b		KY562107	-	KY562375	KY562241
63	185a	40.06 N 9.28 E	KY562108	KY562518	KY562376	KY562242
63	185b		KY562169	-	KY562377	KY562243
64	db15233	40.05 N 9.64 E	KY562109	KY562519	KY562378	-
64	db15234		KY562110	KY562520	KY562379	KY562244
65	db15228	39.98 N 9.63 E	KY562111	KY562521	KY562380	KY562245
65	db15229		KY562112	KY562522	KY562381	-
66	db15224	39.74 N 9.67 E	KY562113	KY562523	KY562382	KY562246
66	db15225		KY562114	KY562524	-	KY562247
67	db15188	39.77 N 9.51 E	KY562115	KY562525	KY562383	KY562248
67	db15194		KY562116	KY562526	KY562384	KY562249
68	184	39.88 N 9.09 E	KY562117	KY562527	KY562385	KY562250
69	183a	39.74 N 9.00 E	KY562118	KY562528	KY562386	KY562251
69	183c		KY562119	KY562529	KY562387	KY562252
69	183d		KY562120	KY562530	KY562295-6	KY562253
69	183e		KY562121	KY562531	KY562388	-
69	330a		KY562122	KY562532	KY562389	KY562254
69	330b		KY562123	KY562533	KY562390	KY562255
70	Ss	39.89 N 8.44 E	EF165022	KY562534	-	-
71	164a	39.55 N 8.57 E	KY562124	KY562535	KY562285-6	-
71	164b		KY562125	KY562536	KY562391	KY562256
72	166	39.43 N 8.50 E	KY562126	KY562537	KY562392	KY562257
73	170a	39.05 N 8.38 E	KY562127	KY562538	KY562393	KY562258
73	170b		KY562128	KY562539	KY562394	KY562259
74	171a	39.19 N 8.77 E	KY562129	KY562540	-	KY562260
74	171b		KY562130	KY562541	KY562395	KY562261
74	171c		KY562131	KY562542	KY562396	KY562262
74	171d		KY562132	KY562543	KY562397	KY562263
74	171e		KY562133	KY562544	KY562398	KY562264
75	350a	39.20 N 8.82 E	KY562134	KY562545	KY562399	KY562265
75	350b		KY562135	KY562546	KY562400	KY562266
76	342a	39.16 N 8.87 E	KY562136	KY562547	KY562287-8	KY562267
76	342b		KY562137	KY562548	KY562401	KY562268
76	342c		KY562138	KY562549	KY562402	KY562269
77	333	39.20 N 8.91 E	KY562139	KY562550	KY562403	KY562270
77	400		KY562140	KY562551	KY562404	KY562271
78	181a	39.54 N 9.34 E	KY562141	KY562552	KY562405	KY562272
78	181b		KY562142	KY562553	KY562406	KY562273
78	181c		KY562143	KY562554	KY562407	KY562274
78	181d		KY562144	KY562555	-	-
78	181e		KY562145	KY562556	KY562408	KY562275
79	345	39.29 N 9.41 E	KY562146	KY562557	KY562410	KY562278
79	172a		KY562147	KY562558	-	KY562276
79	172b		KY562148	KY562559	KY562409	-
79	172c		KY562149	KY562560	-	KY562277
79	7F1		KY562150	KY562561	-	-
79	7F2		KY562151	KY562562	KY562411	KY562279
80	175	39.17 N 9.56 E	KY562152	KY562563	-	KY562280
81	173	39.14 N 9.54 E	KY562153	KY562564	KY562413	-

**Table 2:** GenBank accession numbers of the sequences used in this study to build the phylogeny of the genus *Podarcis*. The references for the sequences obtained from GenBank are reported

Species	*12S*	*nd4*
*Podarcis bocagei*	DQ081064 [61]	DQ081153 [61]
*Podarcis carbonelli*	DQ081065 [61]	DQ081154 [61]
*Podarcis cretensis*	KY561996 ^a^	KY562001 ^a^
*Podarcis filfolensis*	KY561997 ^a^	KJ027796 [58]
*Podarcis gaigae*	AF133444 [126]	KY562002 ^a^
*Podarcis hispanica*	DQ081070 [61]	DQ081171 [61]
*Podarcis guadarramae*	AF469452 [61]	DQ081165 [61]
*Podarcis lilfordi*	KY561998 ^a^	KY562003 ^a^
*Podarcis melisellensis*	AF133448 [126]	KY562004 ^a^
*Podarcis milensis*	AF133449 [126]	KY562005 ^a^
*Podarcis muralis*	KX080575 [44]	KF372393 [60]
*Podarcis peloponnesiaca*	AF133451 [126]	KY562006 ^a^
*Podarcis pityusensis*	KY561999 ^a^	KY562007 ^a^
*Podarcis raffonei*	KY562000 ^a^	KJ027980 [58]
*Podarcis sicula*	KX080574 [44]	KF372035 [60]
*Podarcis taurica*	AF080279 [127]	KY562008 ^a^
*Podarcis tiliguerta* lineage1	KY562020 ^a^	KY562427 ^a^
*Podarcis tiliguerta* lineage2	KY562081 ^a^	KY562492 ^a^
*Podarcis tiliguerta* lineage3	KY562092 ^a^	KY562503 ^a^
*Podarcis tiliguerta* lineage4	KY562146 ^a^	KY562557 ^a^
*Podarcis vaucheri*	HQ898229 [61]	HQ898028 [61]
*Podarcis wagleriana*	DQ017659 [40]	KJ027979 [58]
*Scelarcis perspicillata*	KX080591 [44]	KX081031 [44]
*Teira dugesii*	KX080595 [44]	KX081035 [44]

Numbers in square brackets after GenBank accession numbers refer to publications that generated the cited GenBank data (^a^sequences generated in this study)

**Table 3:** Genetic diversity estimates at *nd4* and *mc1r* loci in *Podarcis* lizards

Species	Species range (km^2^)	*nd4*							*mc1r*						
		N	ns	S	h	Hd (SD)	π (SD)	K	N	ns	S	h	Hd (SD)	π (SD)	K
*P. bocagei* [55–57]	58387	22	661	18.0	18.0	0.978 (0.021)	0.00321 (0.00034)	2.1	12	583	5.0	3.0	0.621 (0.118)	0.00377 (0.00075)	2.2
*P. carbonelli* [55, 56]	4684	25	661	25.0	20.0	0.980 (0.017)	0.00607 (0.00058)	4.0	-	-	-	-	-	-	-
		22	661	23.5	18.1	0.980	0.00610	4.0							
*P. filfolensis* [58]	338	94	661	32.0	27.0	0.900 (0.020)	0.00471 (0.00040)	3.1	130	583	20.0	27.0	0.904 (0.012)	0.00535 (0.00024)	3.1
		22	661	17.4	12.3	0.904	0.00473	3.1	12	583	9.8	7.7	0.899	0.00537	3.1
*P. lilfordi* [59]	<100								92	583	23.0	27.0	0.920 (0.015)	0.00520 (0.00037)	3.0
									12	583	11.6	8.5	0.924	0.00532	3.1
*P. muralis* [60]	1830883	77	661	141.0	55.0	0.987 (0.005)	0.03089 (0.00112)	20.4	162	583	35.0	31.0	0.835 (0.017)	0.00308 (0.00025)	1.8
		22	661	97.5	19.3	0.985	0.03096	20.5	12	583	6.7	6.2	0.832	0.00304	1.8
*P. pityusensis* [59]	741								44	583	10.0	14.0	0.897 (0.022)	0.00416 (0.00031)	2.4
									12	583	7.0	7.4	0.900	0.00423	2.5
*P. tiliguerta* ^a^	32898	148	661	220.0	104.0	0.9936 (0.0017)	0.09994 (0.00191)	65.8	250	583	92.0	148.0	0.9897 (0.0020)	0.00691 (0.00024)	4.0
		22	661	190.2	20.7	0.994	0.10072	66.5	12	583	17.6	11.3	0.989	0.00685	4.0
*P. vaucheri* species complex [55, 56, 61, 62]	305794	87	661	135.0	68.0	0.995 (0.003)	0.03492 (0.00166)	23.1	-	-	-	-	-	-	-
		22	661	94.5	20.9	0.995	0.03558	23.5							
*P. wagleriana* ^b^	23914	101	661	30.0	28.0	0.828 (0.029)	0.00396 (0.00023)	2.6	186	583	13.0	16.0	0.790 (0.020)	0.00289 (0.00021)	1.7
		22	661	12.8	9.9	0.833	0.00398	2.6	12	583	5.9	5.3	0.764	0.00276	1.6

*N* number of sampled gene copies, *ns* number of sites of the alignment used for the calculations, *S* number of segregating sites, *h* number of haplotypes, *Hd* haplotype diversity, *π* nucleotide diversity, *K* average number of pairwise differences, *SD* standard deviation. For the diversity measures S, h, Hd, π and K values calculated under a resampling approach are also reported below values calculated on the original datasets (see text for details). Numbers in square brackets after species names refer to publications that generated the sequence data (^a^sequences generated in this study; ^b^Salvi et al., unpublished sequence data). Species range (extent of occurrence) is based on spatial data from [IUCN, 2016]

Tissue samples were collected as tail tips and stored in ethanol; each individual was then released at the place of capture. Genomic DNA was extracted following standard high-salt protocols [43]. We amplified two mitochondrial gene fragments, 12S rRNA (*12S*) and NADH dehydrogenase subunit four with flanking tRNA^Ser^, tRNA^His^, and tRNA^Leu^ (*nd4*), and two nuclear gene fragments, Melanocortin receptor 1 (*mc1r*) and acetylcholinergic receptor M4 (*acm4*), by polymerase chain reaction (PCR). Primers and PCR protocols used for the amplification of the molecular markers are described in [44].

Additionally, we cloned the *mc1r* PCR products of eight selected heterozygous individuals (showing 4–6 polymorphisms), because for this marker a pilot phasing analysis carried out on a preliminary subset of individuals (*N* = 35) showed high uncertainty of haplotypic phase estimate. PCR products were ligated into pGEM-T Easy Vector Systems kit (Promega) according to the manufacturer’s instructions. The output of the ligation reaction was then transformed into *Escherichia coli* competent cells and grown on standard LB medium with ampicillin/IPTG/X-Gal. For each sample we randomly selected six colonies for sequencing in order to account for PCR/cloning errors resulting from misincorporation of individual nucleotides [45]. We used a conventional blue/white screening to select the 48 positive colonies which were then amplified using universal primers pUC/M13F and pUC/M13R. PCR reactions were carried out in 25 μL volumes containing 1X PCR buffer, MgCl2; 1 mM each dNTP, 2U of GoTaq DNA polymerase (Promega), 0.4 μM each primer and 2 μL of colony DNA. After verification of successful amplification, the inserts were sequenced from both strands with the same primers used for amplification. Purification and sequencing of PCR products and plasmid DNA from positive clones were carried out by Macrogen Inc.

### Data analysis

We used GENEIOUS 6.0 (www.geneious.com) to check electropherograms, calculate consensus sequences, perform multiple sequence alignment, and to annotate both tRNAs in the *nd4* fragment and codon positions in coding regions. Sequence divergence (uncorrected *p-*distance) was assessed using MEGA 6 [46]. Heterozygous sequences within the *acm4* and *mc1r* fragments were phased using PHASE 2.1.1. [47]. Three independent runs were conducted using a model with recombination (−MR0 option), 1000 initial iterations discarded as burn-in, one as thinning interval, and 1000 post-burnin iterations. After monitoring the goodness of fit for each run according to the program’s manual, we accepted haplotype reconstructions which yielded the same result in all of the three runs. Cloned *mc1r* sequences from each selected individual were used to determine the constituent alleles (haplotypes). Cloning-determined haplotypes of these individuals were first compared with corresponding haplotypes inferred by PHASE, in order to assess the inference accuracy, and then used as ‘known phase’ in a further PHASE analysis in order to improve haplotype estimation. For each nuclear gene dataset recombination detection was performed in RDP 4.77 [48] using five different algorithms: BootScan [49], GENECONV [50], MaxChi [51], RDP [52], and SiScan [53].

For each gene we estimated the following summary statistics of genetic diversity using DNASP 5 5.10.01 [54]: number of segregating sites (S), number of haplotypes (h), haplotype diversity (Hd), nucleotide diversity (π), and average number of pairwise differences (K) both overall and for clades defined within the species by phylogenetic analyses. Since DNASP removes entirely sites with missing data, in order to maximize the length of alignments used for diversity calculations we used fully phased haplotype datasets, we excluded sequences with more than 1.5% of missing data, and recoded the few remaining missing data with the most common nucleotide at that site (thus using the information of these sites while retaining the observed level of variation). In order to compare the degree of genetic diversity observed in *P. tiliguerta* with congeneric species, we compiled a synopsis of genetic diversity estimates for *nd4* and *mc1r* loci for other *Podarcis* lizards based on data from previous phylogeographic studies which sampled fairly well each species range [55–62]. Haplotype phases for *mc1r* sequences downloaded from GenBank were resolved for each species separately, using PHASE (100 initial iterations). Retrieved datasets had different sizes, both in terms of number of sites and number of sequences, which may affect comparisons of diversity measures. Therefore, first we trimmed all alignments at the same number of sites, and then we also calculated genetic diversity statistics following the resampling approach described in [60]. Resampling was performed with the aid of a series of scripts written in Python 2.7.1 and taking advantage of DNASP “batch mode” calculations. Tajima’s *D* [63] test were used to assess whether mtDNA and nucDNA sequences of *P. tiliguerta* fitted a neutral model of evolution. *D* values were estimated in DNASP and their significance was assessed through 10,000 coalescent simulations under the hypothesis of population equilibrium and selective neutrality.

We performed a Bayesian evolutionary analysis in BEAST 1.8.0 [64] to estimate phylogenetic relationships among mitochondrial haplotypes and associate a time/age at each node of the phylogeny. BEAST allows the incorporation of the uncertainty associated with phylogenetic estimates, calibration dates and among-lineage variation of substitution rates in a single analysis [64]. In addition BEAST allows sampling the root of the tree by using the molecular clock method [65] without the need of using an outgroup taxon. This is particularly desirable when outgroup rooting is problematic as for *P. tiliguerta* which shows deep branching of mitochondrial lineages with unresolved position within the *Podarcis* phylogeny [24]. One the other hand, for our dataset the choice of a proper tree prior among those available in BEAST is not straightforward as we have an intraspecific dataset where we expect a deep geographic structure [24]. Thus, both a simple coalescent prior [e.g. [66]] which assumes that the individuals analysed are drawn from a single panmictic population – and a multi-species coalescent prior [64, 67] which allows accounting for multiple divergent populations each one following a coalescent process but assume reproductive isolation between them – are not fully appropriate for our data. Therefore, while we acknowledged that available priors are a rough simplification of real situations, we explored the sensitivity of our phylogenetic and dating results to different prior choices and we ran multiple analyses using both priors. Simple coalescent models were implemented in BEAST using the skyline coalescent prior. The multi-species coalescent model was implemented in the *BEAST extension. For both analyses, we implemented the HKY model for the *nd4* coding region and the HKY + G + I for non-coding region (tRNAs +*12S*) according to the best partition schemes and substitution models selected by PARTITION FINDER 1.1.1 [68] under the Bayesian Information Criterion (BIC). We implemented a relaxed uncorrelated lognormal clock model with the normal distribution N (0.0115, 0.00075) for the *nd4* substitution rate prior (parameter ucld.mean). This rate was estimated by [58] for the same *nd4* region used in this study in a calibrated phylogeny of Sicilian *Podarcis* based on the palaeogeographic events associated to the splits between *P. filfolensis* and *P. wagleriana* and between the latter and *P. raffonei* (for a full account on the specific calibration points and methods used see [58]). Similar estimate of substitution rates were previously obtained for *Podarcis* lizards for the same *nd4* gene fragment [55]. BEAST and *BEAST were run twice, with 100 million iterations per run, sampling every 10000 steps. Convergence diagnostic and summary of posterior samples of parameters were assessed in TRACER 1.6 (available at http://beast.bio.ed.ac.uk/Tracer); sampled trees from independent runs were combined in LogCombiner; and Maximum Clade Credibility Trees and Bayesian Posterior Probabilities associated to nodes (BPP) were calculated in TreeAnnotator (burn-in = 25%). We verified consistency of results with different models through additional analyses using unlinked substitution models for the *nd4* codon positions and strict clock models.

In order to identify the main phylogenetic discontinuities along terminal branches of the mitochondrial phylogeny we used the statistical parsimony network approach [69] implemented in the software TCS 1.21 [70]. Subnetworks obtained under the 95% probability criterion for a parsimonious connection have been successfully used as an objective way to identify significant genetic discontinuities in mitochondrial DNA sequence dataset [71].

A preliminary mitochondrial dataset [24] of about 700 base pairs (*12S* and *cytb*) showed two highly divergent mitochondrial lineages in *P. tiliguerta* (maximum *cytb* divergence = 15%) which neither form a monophyletic group nor have a resolved position in the *Podarcis* phylogeny. Therefore we used one representative of each *P. tiliguerta* lineage recovered from our BEAST analyses to infer their phylogenetic relationships within *Podarcis*. In doing so we sequenced 10 *Podarcis* species from the same two fragment used in this study and retrieved Genbank sequences from nine additional *Podarcis* species (Table 2). We designated *Scelarcis perspicillata* and *Teira dugesii* as outgroups based on the mitochondrial phylogeny of Lacertini [44]. We performed a Bayesian analysis in BEAST, using the same settings as in the BEAST analysis of the *P. tiliguerta* dataset but using a Yule process of speciation as tree prior. A Maximum Likelihood (ML) analysis was also performed in Raxml GUI 1.1.3 [72], a graphical front-end for RaxML 7.4.2 [73]. ML searches included 100 random addition replicates and 1000 nonparametric bootstrap replicates, applying the general time-reversible model with gamma model of rate heterogeneity (GTRGAMMA) for both the *nd4* and *12S* partition. To determine if the monophyly of *P. tiliguerta* lineages could be rejected, we used Mesquite 3.03 [74] to generate a tree enforcing the monophyly of *P. tiliguerta* lineages; we then estimated per-site log likelihood values of the best ML tree and the constrained tree in RAxMLGUI and we compared these values using the Shimodaira–Hasegawa (SH) [75] and the approximately unbiased (AU) [76] tests as implemented in CONSEL [77].

We inferred the genealogical relationships between the *acm4* and *mc1r* haplotypes detected in *P. tiliguerta* using the statistical parsimony network approach implemented in TCS. This method is particularly appropriate when few characters for phylogenetic analysis are available due to shallow levels of divergence [78] as we observed in the *acm4* and *mc1r* datasets. Networks were constructed under the 95% probability criterion for a parsimonious connection and represented graphically using the tool tcsBU [79]. Additionally, since *mc1r* sequences are available in GenBank for other *Podarcis* species, we combined them with sequence data from the present study to examine the phylogenetic relationships of *mc1r* haplotypes from different populations and species. For this phylogenetic analysis we used the same *mc1r* datasets used for genetic diversity comparisons plus a few *mc1r* sequences available for two additional species, *P. carbonelli* and *P. sicula* [57, 80].

To investigate the hierarchical structure of the genetic diversity both at mitochondrial and nuclear loci we used the approach implemented in SAMOVA v.2 [81]. This method is based on the analysis of molecular variance (AMOVA) [82] through a simulated annealing procedure and allows defining groups of populations that are genetically homogeneous and maximally differentiated from each other, without the prior assumption of group composition [81]. Samples represented by less than four sequences were not included in the analysis. The 17 mitochondrial monophyletic sublineages previously identified by the phylogenetic analyses were treated as populations in our SAMOVA analyses; accordingly mtDNA sequences from neighbouring localities belonging to the same sublineage were pooled and geographic coordinates for each population were calculated as the geographic centroid of member localities. We ran the analysis both enforcing the geographical homogeneity of groups and without constraint for the geographic composition of the groups in order to investigate both spatial and non-spatial structure of the genetic dataset. SAMOVA was run three times for each value of predefined number of groups (*K*) using the TN + G substitution model, 100 random initial conditions and 10,000 iterations. For each gene we tested genetic structures from *K* = 2 until increasing *K* generated genetic structures with non-significant fixation indexes. Additionally, we explored patterns of genetic differentiation exhibited by nuclear genes by performing AMOVA using mtDNA-defined partitions. These analyses were conducted using ARLEQUIN 3.5.2.2 [83].

In order to explore whether nuclear genetic divergence of populations correlate with geographic distances we carried out Mantel tests and reduced major axis (RMA) regression analyses using IBDWS 3.23 [84]. Genetic differentiation was calculated both as simple F_ST_ (based on haplotype frequencies only) and Phi_ST_ (also accounting for haplotype divergence estimated as K2P distance) and significance was assessed through 1000 random permutations.

Finally, in order to assess whether past habitat suitability may have impacted the geographic distribution of *P. tiliguerta* and its genetic diversification, we performed species distribution modeling (SDM) under current and Last Glacial Maximum (LGM; ~21 thousands years ago, 21 kya) bioclimatic envelops using the maximum-entropy algorithm implemented in MaxEnt 3.3.3e [85]. We collected 1075 presence data of *P. tiliguerta* from personal observations (including sampled localities), previous literature and public databases. In order to correct for potential sampling biases in the distribution records [86], we selected 480 points of species occurrence with a minimum distance of 3 Km. The bioclimatic layers were downloaded from the WorldClim database website (www.worldclim.org). For the LGM prediction we used data from two different general circulation models (CCSM and MIROC). We built the models with a set of five variables that were not strongly correlated with each other (Pearson’s correlation coefficient, r^2^ < 0.80) and that we deemed as biologically significant for *P. tiliguerta*. Selected variables were: temperature seasonality (BIO4), temperature annual range (BIO7), mean temperature of the driest quarter (BIO9), annual precipitation (BIO12) and precipitation seasonality (BIO15). We ran Maxent with autofeatures, selecting at random 70% of the presence records as training data and 30% as test data for each species. We tested the quality of the models by calculating the area under the curve (AUC) of the receiver operated characteristics (ROC) plots [87]. We built models with an increasing regularization parameter β and between them we selected the model that best fit the data under the Akaike Information Criterion (AIC) with ENMTools 1.3 [88].

## Results

The concatenated mitochondrial (mtDNA) dataset (*12S* + *nd4*) included 171 sequences and 1233 aligned positions (149 individuals sequenced for both fragments, 22 individuals sequenced only for the *12S*). For three individuals we were only able to obtain nuclear sequences. The nuclear (nucDNA) datasets consisted of 250 (phased) sequences for *mc1r* (614 positions) and 216 for *acm4* (401 positions) and had no missing data. Multiple sequence alignment did require four gap positions in the tRNAs and *12S* regions; coding regions (*nd4*, *acm4,* and *mc1r*) did not require gap positions and their translation into amino acid sequences contained no stop codons. We obtained 43 *mc1r* sequences from the 48 positive colonies and within them we observed 57 instances of nucleotide misincorporation (0.21% of the total nucleotides) (Additional file 1: Table S1). When comparing *mc1r* haplotypes determined by cloning and inferred by PHASE they were identical at 33 of the 39 heterozygote sites (85%). The recombination tests applied in RDP did not find statistically significant evidence for recombination in any of the nuclear genes.

The overall level of sequence variation in the mitochondrial and nuclear loci was high (mtDNA: S = 285, h = 110, Hd = 0.995 ± 0.001, π = 0.07264 ± 0.00151, K = 82.442; *acm4*: S = 66, h = 105, Hd = 0.958 ± 0.008, π = 0.00818 ± 0.00037, K = 3.280; *mc1r*: S = 96, h = 157, Hd = 0.991 ± 0.002, π = 0.00688 ± 0.00023, K = 4.226). A summary of genetic diversity estimates and neutrality tests per locus for each lineage and island is given in the Additional file 2: Table S2. Genetic diversity estimated as S, H, and K, was higher in *P. tiliguerta* compared to congeneric species (Table 3). Significantly negative Tajima’s *D* values (excess of rare alleles), indicative of population expansion, purifying selection, or genetic hitchhiking, were found in *acm4* (*D* = −2.121, *P* < 0.001) and *mc1r* (*D* = −2.222, *P* < 0.001). Non-significant *D* were found overall in mtDNA (*D* = 1.999, *P* = 0.979) and in all mtDNA lineages.

**Fig. 2:**
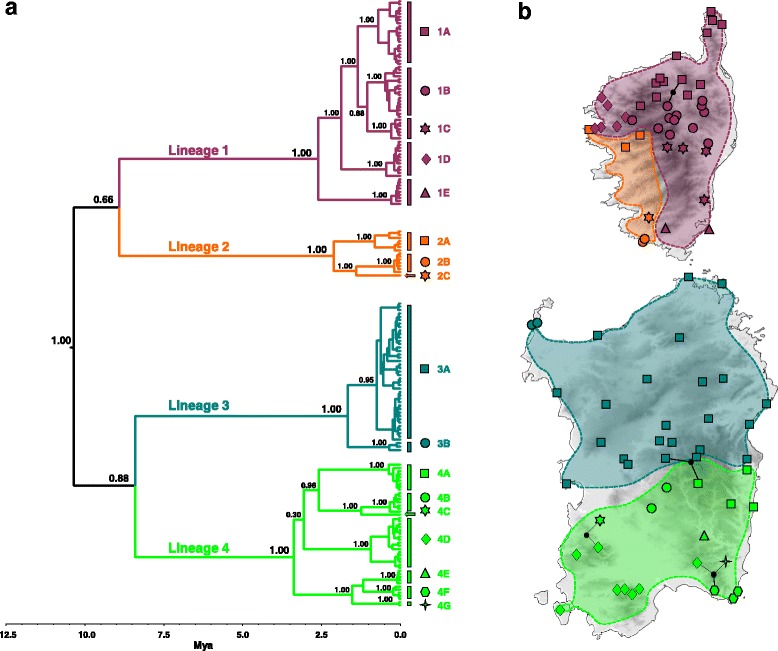
Bayesian time tree depicting the phylogenetic relationships among combined mitochondrial sequences (*12S* and *nd4*) of *Podarcis tiliguerta* (**a**). Bayesian Posterior Probabilities are reported in correspondence of the nodes of the main lineages (Lineage 1–4) and sublineages (1A-1E, 2A-2C, 3A-3B, 4A-4G) and the tree scale showed the estimated times to their most recent common ancestor (TMRCA) in million years (see text for further details and highest posterior density intervals). The map shows the geographic distribution of mitochondrial lineages and their putative ranges (**b**)

Bayesian phylogenetic trees inferred in BEAST (Fig. 2a) and *BEAST (not shown) based on mtDNA sequences showed four main lineages which are well supported (BPP = 1.00) and have geographic coherence: Lineage one including individuals from north and east Corsica, Lineage two from west Corsica, Lineage three from north Sardinia and Lineage four from south Sardinia. Relationships between the four main lineages are not statistically supported (BPP ≤ 0.88). Within these lineages, haplotypes clustered in 17 well supported sub-lineages (1A-1E, 2A-2C, 3A-3B, 4A-4G; all BPP = 1.00) with a strict phylogeographic association (i.e. formed by geographically proximate individuals). The same 17 sub-lineages were recovered as distinct sub-networks in the statistical parsimony analysis (Additional file 3: Figure S1).

Average genetic distance between mtDNA lineages of *P. tiliguerta* was 11.65% at the *nd4* and 5.23% at the *12S* fragment (ranging from 11.1 to 12.4% for *nd4* and from 3 to 6.5% for *12S*). Within lineages average genetic distance was 2.65% at the *nd4* and 1.07% at the *12S* fragment (*nd4*: 1–4.2%; *12S*: 0.6–1.3%).

The *12S* substitution rate estimated by BEAST based on the *nd4* rate prior implemented was 0.53% (0.37–0.69 95% HPD) per million years which is in agreement with substitution rates calculated for lacertid lizards for the same *12S* gene region (0.55% ± 0.13% SD) in [89], based on a phylogeny calibrated using seven biogeographic calibration points. This correspondence provides further justification for the *nd4* rate used in our molecular clock model.

According to our mitochondrial clock model the main cladogenetic events within *P. tiliguerta* occurred during the Miocene. The time to the most recent common ancestor (TMRCA) of the main lineages was estimated in BEAST at 10.3 million years ago (mya) (Fig. 2a) with an associated 95% highest posterior density (95% HPD) interval of 7.9–13.2 mya. For the Corsican lineages (Lineage 1 and 2) the TMRCA was estimated at 8.9 mya (95% HPD: 6.5–11.5) and for the Sardinian lineages (Lineage 3 and 4) at 8.4 mya (95% HPD: 6.1–11.1 mya), although these nodes received low posterior probability. The TMRCAs estimated for each main lineage are placed from the Early Pliocene to Early Pleistocene (3.4–1.7 mya; 95% HPD intervals: 4.3–0.6 mya) and their diversification in 17 sub-lineages bounds the period from the Late Pliocene to Early Pleistocene (3.1–1.3 mya; 95% HPD intervals: 3.9–1.0 mya). TMRCA estimates of the main lineages and the time of splits of the 17 sub-lineages calculated in *BEAST under the multi-species coalescent model (results not shown) are virtually identically to those calculated in BEAST. On the other hand, the TMRCA of the main lineages estimated in *BEAST (8.4 mya; 95% HPD: 6.9–10.2) is more recent compared to estimates from the BEAST analysis reported above.

The four mitochondrial lineages of *P.tiliguerta* represent deep branches in the mitochondrial phylogeny of *Podarcis* and are included in a basal polytomy (Additional file 4: Figure S2), although the hypothesis that they are monophyletic cannot be rejected (SH-test, *P* = 0.291; AU-test, *P* = 0.279).

**Fig. 3:**
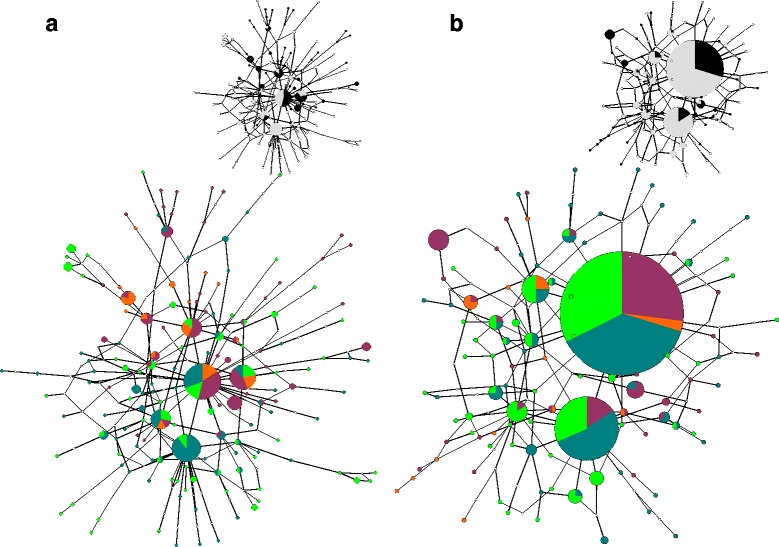
Statistical parsimony networks showing the genealogical relationships between the *mc1r* **a** and *acm4* **b** haplotypes. Haplotypes are represented by circles with size proportional to their frequency; small white circles represent ‘missing’ or extinct haplotypes. Nuclear haplotype are coloured according to the mitochondrial lineage to which each individual belongs: the large networks map the four main lineages whereas the small networks in the top of panel **a** and **b** map Corsican (*black*) *versus* Sardinian (*white*) lineages

Nuclear genealogies showed high haplotype diversity but shallow divergence and a lack of phylogeographic structure (Fig. 3). Both the *mc1r* and the *acm4* networks showed a reticulated pattern of relationships between closely related haplotypes (maximum number of mutational steps: ten for *mc1r* and eight for the *acm4*). Nuclear haplotypes at both loci were broadly shared between individuals belonging to distinct mtDNA lineages and between Corsican and Sardinian individuals (Fig. 3).

Phylogenetic relationships between *mc1r* haplotypes inferred from 946 phased sequences from nine *Podarcis* species show that, although separated by a few mutations, almost all haplotypes inferred (284, equal to 98.7% of the total) were species-specific (Additional file 5: Figure S3). Haplotypes carried by conspecific individuals tend to cluster together in the phylogenetic network, although a pattern of strict reciprocal monophyly between species was not observed. Four transpecific haplotypes (1.3% of the total) are shared between the species *P. lilfordi, P. pityusensis* and *P. tiliguerta*, and probably reflects a recent common history of these species.

**Fig. 4:**
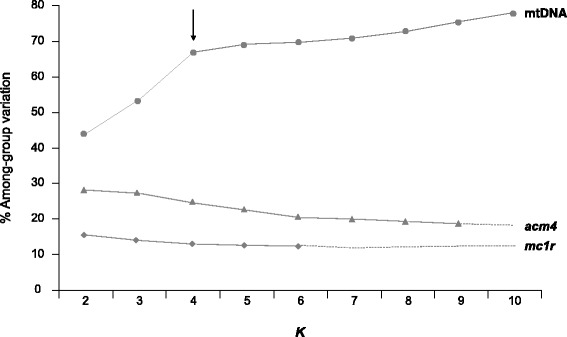
Summary of results of the spatial analysis of molecular variance (SAMOVA) analyses of the sampled populations of *Podarcis tiliguerta* for each locus. The percentage of variation explained by the among-group level of variation is reported for the best-clustering option at each pre-defined value of *K* (the number of groups). The *arrow* shows the best clustering option obtained for the mtDNA dataset, whereas SAMOVA analyses based on nuclear (*mc1r* and *acm4*) dataset do not allow identifying a population grouping best explaining the data. *Dashed lines* represent values of *K* for which fixation indexes were not significant

The hierarchical genetic structure of *P. tiliguerta* inferred by SAMOVA analyses strictly correspond to the geographic partition of genetic lineages (or lack thereof) found by phylogenetic analyses (Fig. 4). Based on mtDNA data SAMOVA identified the four groups of populations corresponding to the main mtDNA lineages as the grouping option which best explains the among-group partitioning of molecular variance (*K* = 4; *F*_CT_ = 0.67; *P* < 0.001). Consistent SAMOVA results were obtained either enforcing or not the spatial association between populations within groups. The subdivision into four main lineages explains 67% of the overall genetic variation compared with 26.8% due to differences between populations within lineages and 5.86% due to differences within populations. All variance components were highly significant providing evidence of a strong genetic structure (*P* < 0.001). SAMOVA analyses based on nucDNA data either enforcing or not the geographic homogeneity of groups showed a lack of genetic and geographic structure in nuclear genes. Partitioning populations in any number of groups explained less than 28 and 15% of the total amount of genetic variation for *acm4* and *mc1r* respectively, whereas more than 65 and 71% of variation (for *acm4* and *mc1r* respectively) was accounted by differences within populations. In most cases, and in particular for *K* = 2–4, all groups except one were defined by a single population. In all AMOVA analyses carried out on *acm4* and *mc1r*, using mtDNA-defined partitions, 92–97% of the total genetic variation was explain by differences within populations whereas higher hierarchical level only explained 1–7% of the genetic variation (Additional file 6: Table S3).

Mantel tests and scatterplots of pairwise genetic and geographic distances between populations showed no support for a pattern of isolation by distance at nuclear loci (Additional file 7: Figure S4). At the *acm4* locus the association between either F_ST_ or on Phi-_ST_ genetic distance and geographic distance was non-significant (F_ST_: Z = 2525111.7735, r = 0.0733, one-sided *P* = 0.144; Phi_ST_: Z = 6740977.5934, r = 0.0754, one-sided *P* = 0.130). At the *mc1r* locus the association between F_ST_ and geographic distance was not significant (Z = 3915308.0029, r = 0.0847, one-sided *P* = 0.124) whereas Phi_ST_ and geographic distance showed a weak but significant association (Z = 11681710.8232, r = 0.2539, one-sided *P* < 0.001) explaining less than 1% of the genetic variance at this locus (R^2^ = 0.064). However, like the previously generated scatter plots, the plot of the *mc1r* Phi-_ST_ distance versus geographic distance failed to reveal a positive and monotonic relationship, showing instead a wide degree of scatter of genetic distance values over all geographic distances values (Additional file 7: Figure S4).

**Fig. 5:**
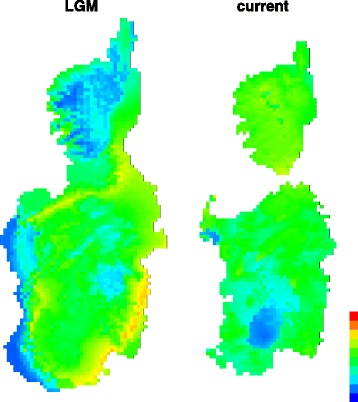
Species distribution models (SDM) based on bioclimatic variables for *Podarcis tiliguerta* under the present-day conditions (current) and the Last Glacial Maximum (LGM; ~21 thousands years ago, 21 kya). Warmer colours show areas with higher bioclimatic suitability

The AUC values of the SDM built using the five selected variable model were >0.7 for the training and the test data, indicating a good performance of the model. Projections of the model over LGM bioclimatic conditions using the MIROC and the CCSM databases produced comparable suitability areas, therefore only the MIROC model is shown (Fig. 5). Current species distribution model showed high bioclimatic suitability for *P. tiliguerta* across Corsica and Sardinia except for the Campidano plain in south Sardinia, which fits the known continuous distribution pattern of the species. Suitable bioclimatic conditions were widespread also during the LGM especially in the lowlands made available by marine regression such as in the wide expanse of land connecting Corsica and Sardinia. Lower suitability was apparent in mountainous regions of Corsica and western Sardinia.

## Discussion

### An insular hotspot of genetic diversity within *Podarcis tiliguerta*

The Tyrrhenian wall lizard *Podarcis tiliguerta* shows unparalleled high genetic diversity compared either to island endemics or to continental species. Both at mitochondrial and nuclear loci, the amount of segregating sites, nucleotide diversity, and mean number of pairwise differences are many times greater in *P. tiliguerta* than in continental-island endemic species such as *P. wagleriana* or in species widespread across the Western Palaearctic such as *P. muralis* and the *P. vaucheri* species complex (Table 3). The values of mitochondrial nucleotide diversity found in *P. tiliguerta* are the highest observed in *Podarcis* lizards and among the highest observed in reptiles at the same loci. Such a vast nucleotide diversity is reflected in a complex phylogeographic pattern. Across the relatively small range of *P. tiliguerta* we found a geographic mosaic of 17 allopatric haplogroups sharply divided into four main mitochondrial lineages. The genetic divergence between these four lineages exceeds the mitochondrial differentiation usually observed between *Podarcis* species and in general between reptile species [24]. This high genetic diversity pattern is consistent with previous studies using mitochondrial [24, 40, 41] and nuclear [21] markers. In particular [21] found high level of heterozygosity within *P. tiliguerta* at allozyme loci with a value (*H*_*o*_ = 0.066) that exceeds the average heterozygosity estimated in *Podarcis* species (*H*_*o*_ = 0.053; [90]) and in reptile species (*H*_*o*_ = 0.047; SD = 0.028 [91]).

The exceptional level of genetic diversity observed within *P. tiliguerta* is puzzling in view of its insular condition and continuous distribution across a restricted range (see Table 3 for species range size). Furthermore, the conditions for the long term persistence of such diversity have not been particularly more stable within the range of *P. tiliguerta* as it was for other Mediterranean islands compared to neighbouring continental areas [e.g. [58]]. Indeed, during the last glaciation northern Corsica was substantially influenced by polar air [92], and extensive glaciers formed on the central mountain chain during Pleistocene glacial phases [93]. Island endemics have long been considered as homogenous entities formed by a single panmictic population with size correlated to island size [94–96]. Likewise, *P. tiliguerta* is currently listed as Least Concern in the IUCN list, based on ‘its wide distribution and presumed large population’ [97]. The high genetic diversity, phylogeographic complexity and intra-island differentiation found within *P. tiliguerta* clearly challenges these simplified assumptions. Similar findings stand out from recent phylogeographic studies on Corsican Sardinian endemic vertebrates [13, 20, 32] as well as on other insular systems [e.g. [98, 99]] indicating that the complexity of evolutionary histories which took place in environmentally complex islands such as the Tyrrhenian islands engendered a wealth of diversity comparable to or even higher than that observed in continental settings.

### Decoupled mito-nuclear histories at the root of the genetic diversity hotspot

Multilocus genetic variation in *P. tiliguerta* shows a profound discordance in spatial and phylogenetic patterning between mitochondrial and nuclear genomes. Two highly divergent mitochondrial lineages occur within each Corsica and Sardinia (Fig. 2), whereas nuclear variation is not phylogenetically (Fig. 3) or geographically (Fig. 4) structured. Under a neutral model of molecular evolution the mtDNA phylogeography of *P. tiliguerta* tells a very different story from nucDNA.

Divergence time estimates placed the coalescence of the Corsican and Sardinian mitochondrial lineages back to the Late Miocene (8–10 mya). Within-island divergence both in Corsica and Sardinia would have started accordingly somewhat later (7–9 mya), although the uncertainty over the monophyly of Corsican and Sardinian populations suggests that such postulation requires caution (Fig. 2; Additional file 4: Figure S2). The primary split between the biotas of Corsica and Sardinia occurred during the disjunction of the Corsica-Sardinia microplate which started 15 mya and was completed by 9 mya [14, 15, 100]. While such a palaeogeographic scenario may explain the onset of the between-islands divergence of mitochondrial lineages, on the other hand, Corsica and Sardinia have not remained geographically independent since their first separation. Further connections between them occurred during the Messinian Salinity Crisis (5.7–5.3 mya) [16, 101] and repeatedly in the Pleistocene at each ice age, from 2.58 mya up to 12 thousands years ago (12 kya) [18, 19]. Especially during the Pleistocene glaciations, faunal exchange between the two Islands has been intense, as documented in the other endemic lizards of Corsica and Sardinia, *Archaeolacerta bedriagae* [12, 27] and *Algyroides fitzingeri* [31], but also in other thermophilic endemic taxa (*Hyla sarda* [30]; *Euleptes europaea* (Salvi et al. unpublished data); see also preliminary data on *Discoglossus sardus* in [11]). Palaeogeographic data and results from SDM analyses (Fig. 5) suggest that also for *P. tiliguerta* the wide lowland area connecting Corsica and Sardinia during glaciation-induced marine regressions has offered suitable habitat and ample dispersal avenues between the two islands. If on one hand we have little evidence that the transitory separations between Corsica and Sardinian may have triggered the vicariance between the *P. tiliguerta* lineages of each island, on the other hand the geologic evolution of Corsica and Sardinia is clearly not associated to the ancient (Miocenic) intra-island divergence estimated between west and east Corsican (Lineage 1 and 2) lineages and between north and south Sardinian (Lineage 3 and 4) lineages. Deep phylogeographic partitions have been found in north Corsica, but not in Sardinia, for the endemic lizard *A. bedriagae* [12] and the Corsican newt *Euproctus montanus* [13] as well as in the snail *Solatopupa guidoni* [29]. While a possible association with an exceptionally dry period during the Pliocene may explain the allopatric divergence between the newt lineages [13], to date we still have no clues on possible historical (environmental) barriers which may have driven intra-island diversification in the former species.

Conversely, the pattern of genetic variation of nuclear markers is consistent with a more recent origin of the observed diversity and with a biogeographic scenario of high inter-island and intra-island connectivity. At both nuclear loci analysed we observed shallow divergence and extensive allele sharing between and within islands. This pattern may arise as a result of long-term demographic stability and absence of significant structuring processes in the recent evolutionary history, but this seems not to be the case for *P. tiliguerta*. Indeed, isolation by distance (IBD) analyses indicate a lack of regional equilibrium between genetic drift and gene flow at nuclear loci. The patterns of correlation between genetic and geographic distances (Additional file 4: Figure S4) strictly match the case III reported in [102], whereby genetic drift is more influential than gene flow. This pattern is expected if conditions conducive to dispersal have not been stable across the region for a long enough period of time for a migration–drift equilibrium to be established, such that at a regional level the species is fragmented (or has been recently fragmented) into small isolated populations [102]. Allozyme data on *P. tiliguerta* [21] also showed population fragmentation and extensive haplotype sharing, with a lack of alternatively fixed alleles between and within Corsica and Sardinia. Interestingly [21] found a latitudinal cline pattern of variation in the three loci displaying the larger allele frequency differences among populations (*Idh-1*, *Gapd*, and *Gpi*) and a significant IBD pattern in the overall allozyme frequencies. While it is difficult to disentangle IBD and clinal patterns of allozyme variation as the sampling of this study is structured along a north–south axis, such a latitudinal pattern lines up with changes in local bioclimatic regimes across the species range, leading to the hypothesis of a possible role for local adaptation in shaping allozyme frequencies and differentiation between populations [21].

### Looking for the source of mito-nuclear discordance

Mito-nuclear discordance is a common phenomenon in animal systems [103], although the spatial and temporal degree of the discordance observed in *P. tiliguerta* is uncommon in literature (but see e.g. [104]]). In many cases it has been possible to conciliate the observed differences in spatial and phylogenetic patterns between mitochondrial and nuclear markers simply accounting for their different mode of inheritance and rates of evolution. Indeed, effective population size of the biparentally-inherited diploid nuclear genome is four times larger than the maternally-inherited haploid mitochondrial genome, which also displays higher mutation rates than the nuclear genome. Therefore, under neutral evolution, historical isolation between populations is readily imprinted in mitochondrial genomes in the form of allopatric genealogical clusters whereas more time is necessary for nuclear genealogies to be sorted (e.g. [105]). This scenario has been invoked to explain dissimilar mitochondrial and nuclear genealogies (including *nd4* and *mc1r* respectively) in other *Podarcis* lizards such as *P. fiflolensis* [58], *P. muralis* [60] and the Iberian and North African species complex [106]. In these cases, further studies using nuclear markers with higher mutation rates (microsatellites) recovered the same groups identified by mitochondrial markers [107–109], corroborating the hypothesis of recent divergence with incomplete lineage sorting at some nuclear loci such as *mc1r*. On the other hand, in the case of *P. tiliguerta* the mitochondrial lineages have been independently evolving for as much as 7–13 million years and the genetic divergence observed between them is at the level of (or higher than) comparisons between distinct *Podarcis* species (see also Additional file 4: Figure S2). In these circumstances we may expect some level of sorting at nuclear loci between lineages as we actually observed at the *mc1r* when examining the phylogenetic relationships between haplotypes from different populations and species (Additional file 5: Figure S3).

If we assume that the deep phylogeographic breaks imprinted in the mitochondrial genome of *P. tiliguerta* are indicative of historical barriers to gene flow among populations, and based on previous studies on *Podarcis* [59, 80] we exclude a role of direct selection in shaping nuclear sequence variation, an alternative hypothesis to explain mito-nuclear discordance could be a scenario of allopatric divergence followed by secondary contact, with nuclear introgression mediated by male-biased asymmetries in dispersal or mating. Male-biased dispersal is unlikely to be the driver of such a pattern in *P. tiliguerta* as dispersal in *Podarcis* is not sex-biased and it would be in any case unrealistic to assume, under extensive gene flow at neutral nuclear loci, a complete lack of female dispersal over a few kilometres along phylogeographic breaks of >100 km during millions of years (see locations 21–22, 25–26 in Corsica or 62–63 and 64–65 in Sardinia, Figs. 1 and 2). On the other hand, strong asymmetries in male competitive ability and mating success, between distinct lizard lineages, may result in asymmetric hybridisation upon secondary contact [110]. While et al. [108] provided compelling evidence for such a scenario in the common wall lizard *P. muralis* and showed how allopatric divergence in sexually selected traits via male competition may determine asymmetric nuclear introgression following secondary contact, with replacement of nuclear characters of the sub-dominant lineage. In the case of *P. muralis* nuclear clines at neutral loci were shifted about 50 km compared to the mitochondrial break, and phenotypic clines (indicative of nuclear loci under sexual selection) were shifted even further (100–200 km). These kind of distances translated into the insular setting of Corsica and Sardinia may explain the replacement of sub-dominant lineages at nuclear loci and the maintenance of a sharp mitochondrial break within each island. However what makes this scenario less likely for *P. tiliguerta* is that these processes should have acted between islands as well, so that mitochondrial lineages should have been fixed at the zones of secondary contacts whereas one nuclear lineage would have replaced the others at the range-wide scale. An additional hypothesis to consider would be that of ghost mitochondrial DNA lineages corresponding to the remnants of former, now extinct species inhabiting the same regions which nuclear background was completely admixed. This scenario has been reported in other *Podarcis* species such as *P. liolepis* and *P. hispanica* [111]. However, this scenario seems highly farfetched for *P. tiliguerta*, since we would be in the presence of at least three such “ghosts”, which seems unlikely – particularly on an island setting. Moreover these scenarios are all hinged on allopatric divergence (e.g. a glacial refugia model in *P. muralis* [60]) and in the case of *P. tiliguerta* we have little biogeographic evidence for an ancient and prolonged vicariance of populations within and between islands, as discussed above.

Deep mitochondrial divergence can arise even in the absence of long-term barriers to gene flow as a result of stochastic and selective processes. Simulations show that strong phylogeographic structure at uniparental loci can form by chance in a continuous population, when dispersal and population size are low, due to the stochastic nature of the coalescent process [112–114]. While conditions for stochastic mtDNA lineage sorting are not met by *P. tiliguerta* (very low dispersal distances and population sizes), further studies have demonstrated that a small amount of selection for local adaptation dramatically increases the range of conditions – i.e. large population size and low to moderate dispersal - under which mitochondrial phylogeographic breaks can arise. Under this model a strong and stable mitochondrial structure can arise under rather weak selection and moderate dispersal in a continuously distributed species with gradual environmental variation [115]. These conditions and the expected pattern match rather well the case of *P. tiliguerta*. This scenario implies no long-term vicariance between populations, highly divergent mitochondrial clades that are geographically localized and unrelated to nuclear variation, and which have much longer coalescent time compared to neutral evolution [115]. This may explain why the origin and the persistence of mitochondrial partitions observed in *P. tiliguerta* do not fit the geological evolution of Corsica and Sardinia and in particular the historical geographic continuity and ecological connectivity of populations between and within islands. It may also account for the lack of association between mitochondrial and nuclear variation and the surprisingly deep divergence times estimated for the mitochondrial lineages under neutral expectations. Weak and/or not very recent selection may explain why selective neutrality tests failed to detect departure from neutrality in the mtDNA dataset, as these tests have been shown to have very low statistical power in such conditions [116].

There is increasing awareness and empirical evidence of selection on mtDNA due to the crucial role in metabolism of mtDNA encoded proteins and since they are part of a perfectly linked group that undergo a high mutation rate [reviewed in e.g. [115, 117, 118]]. A growing number of studies in animal systems showed that cases of striking mito-nuclear discordance can be explained by an association of mitochondrial variation with environmental variables such as climatic gradients [e.g. [119–122]]; see also [123]]. This indicates that geographically structured mtDNA diversity may reflect selective optima across environmental gradients rather than reliably track the species’ evolutionary history. The data collected in this study are not suitable to rigorously test the hypothesis of environmental selection on mtDNA and to understand the mechanisms through which putative female-linked selection operates. These may include selection on one or more mtDNA encoded proteins, W-linked genes, or at nuclear-encoded genes which are functionally related to mitochondrial (e.g. for proteins involved in the oxidative phosphorylation or related to mitochondrial metabolism). While much research will be needed to further investigate these aspects (including an exhaustive sampling across environmental gradients and a genomic-wide screening on mitochondrial, autosomal and sex-linked loci), preliminary evidence suggests that cyto-nuclear adaptation is a possible mechanism shaping genetic variation in *Podarcis tiliguerta* and in general in Corsican-Sardinian lizards. Indeed, [21] suggested that the latitudinal clinal pattern observed in *P. tiliguerta* at three highly heterogeneous allozyme loci (*Idh-1*, *Gapd*, and *Gpi*) was associated with local bioclimatic regimes. In the codistributed rock lizard *A. bedriagae,* [27] provided evidence for a significant association between the (non-clinal) variation at the *Idh-1* locus and annual temperature and precipitation. Some of these enzymes such as the IDH have a close functional relationship with mitochondrial metabolism. Thus, these preliminary findings point to the interesting possibility that local adaptation leading to phylogeographic structure could influence both mitochondrial genes and nuclear genes functionally related to mitochondrial genes. This line of research appears particularly promising on Corsican Sardinian endemics for several reasons: (i) the well delimited and restricted spatial scale of this insular setting [19]; (ii) the availability of previous phylogeographic assessment and well known distribution of the species [12, 13, 20–34]; (iii) the growing number of cases of deep within-islands mitochondrial divergence that has been observed in the absence of a current or historical barrier to gene flow [12, 13, 29]; this study]; and (iv) preliminary evidence of association of allozyme genetic variation with climatic variables [21, 27]. Thus, Corsican-Sardinian endemic lizards appear a promising model for testing how and whether selection has been shaping patterns of within-species diversity in such a biodiversity hotspot.

### Taxonomic and conservation implications

Based on the high value of genetic divergence observed between mitochondrial lineages of *P. tiliguerta*, it has been suggested that this species may represent a species complex [24]. Interpreting deep mitochondrial divergence as indicative of speciation would assume that mitochondrial lineages of *P. tiliguerta* will be the result of long-term barriers to gene flow under neutral evolution; an assumption which received little support in our study. Despite mitochondrial structure, both this study and [21] showed that nuclear variation is shared between and within Corsica and Sardinia with a lack of alternatively fixed alleles. Also morphological data on *P. tiliguerta* showed high variability with a north–south cline of variation across the main islands rather than a sharp transition between geographic units [124]. Bruschi and colleagues [125] based on 11 pholidotic characters failed to identify diagnostic characters between and within Corsican and Sardinian populations, although their sample was mainly composed by micro-insular populations (about 90% of the populations studied) from satellite islets surrounding Corsica and Sardinia, hence of little information as regards range-wide patterns. Therefore, at the moment we have no evidence of the existence of long-term barrier to gene flow within *P. tiliguerta* and convincing conclusions on the taxonomic value of the observed mitochondrial lineages can only be obtained after a fuller understanding of the evolutionary processes that led to the observed phylogeographic pattern.

On the other hand, whether the distinct phylogeographic groups observed within *P. tiliguerta* underline long-term isolation or local adaptation they indicate that this species does not represent in any case a single management and conservation unit. Therefore, each lineage should be individually targeted when setting strategies for the long-term conservation of *P. tiliguerta* and of its evolutionary potential.

## Conclusions

Our study on the multilocus genetic variation of *Podarcis tiliguerta* reveals surprising levels of genetic diversity underlining a complex phylogeographic pattern with a striking example of mito-nuclear discordance. These findings have considerable implications for the taxonomy and conservation of *P. tiliguerta* as well as for our understanding of the processes involved in the evolution of biodiversity hotspots within Mediterranean islands. Neutral models based on long-term vicariance provide unparsimonious explanations for the deep phylogeographic breaks observed in mtDNA within and between islands, both in this species as well in other Corsican-Sardinian endemics. Growing empirical evidence suggests a possible role for local adaptation along a smooth environmental gradient underlying the origin and persistence of highly divergent, geographically localized, mitochondrial groups found in endemic amphibian and reptiles as well as of their geographic patterns of nuclear variation. These hypotheses could be thoroughly tested taking advantage of genomic resources. In this respect, this study represents one more step in our long-term aim to fully understand the evolutionary mechanisms underlying the rising of the Mediterranean diversity hotspot.

## Additional files

Additional file 1: Table S1. Summary results of haplotype phasing based on data from cloning. For each individual heterozygote sites are highlighted in green and the corresponding sequence of each replicated colony is reported. Nucleotide states in red font included in grey boxes indicate instances of nucleotide misincorporaitons (PCR/replication errors) in colonies and were solved following a parsimony approach. (PDF 103 kb)
Additional file 2: Table S2. Summary statistics of molecular diversity and neutrality tests for each locus and each mitochondrial clade within *Podarcis tiliguerta*. N: number of sampled gene copies; ns: number of sites of the alignment used for the calculations; S: number of segregating sites; h: number of haplotypes; Hd: haplotype diversity; π: nucleotide diversity; K: average number of pairwise differences; *D*: Tajima’s *D* values (1989). Significance values for the Tajima’s *D* tests based on 1000 coalescent simulations are shown next to the values (ns: *P* ≥ 0.05; *: 0.01 ≤ *P* < 0.05; **: 0.001 ≤ *P* < 0.01; ***: *P* < 0.001). (PDF 158 kb)
Additional file 3: Figure S1. Results of statistical parsimony network analyses based on mtDNA data. Sub-networks recovered under the 95% limit for a parsimony connection. Haplotypes are represented by circles with size proportional to their frequencies, coloured according to the main mtDNA lineages and labelled according to the sublineages recovered in the phylogenetic analysis (see text for more details). Small white circles represent ‘unsampled or extinct haplotypes. (PDF 86 kb)
Additional file 4: Figure S2. Bayesian phylogenetic tree of Podarcis based on mitochondrial sequences (*12S* and *nd4*). Bayesian Posterior Probabilities values > 0.90 are reported above the nodes; bootstrap values of the Maximum Likelihood analysis > 50 are reported below the nodes. (PDF 72 kb)
Additional file 5: Figure S3. Statistical parsimony network showing the genealogical relationships between the *mc1r* haplotypes inferred for nine *Podarcis* species based on 946 sequences retrieved from Genbank [57–60, 80], generated in this study (*P. tiliguerta*) and unpublished (*P. wagleriana;* Salvi et al. in prep.). Haplotypes are represented by circles with size proportional to their frequency; small white circles represent ‘missing’ or extinct haplotypes. (PDF 104 kb)
Additional file 6: Table S3. Result of analyses of molecular variance (AMOVA) based on partitions defined by mtDNA variation of *Podarcis tiliguerta*. ns: *P* ≥ 0.05; *: 0.01 ≤ *P* < 0.05; **: 0.001 ≤ *P* < 0.01; ***: *P* < 0.001. (PDF 96 kb)
Additional file 7: Figure S4. Patterns of correlation between genetic distances and geographic distance at nuclear loci. Scatterplots of the genetic distances (F_ST_ and Phi_ST_) versus geographic distances (km) among population pairs of *Podarcis tiliguerta* at the loci *acm4* and *mc1r*. The reduced major axis (RMA) regression lines are shown. (PDF 347 kb)

## Abbreviations

*acm4*: acetylcholinergic receptor M4
BPP: Bayesian Posterior Probabilities
BPP: Bootstrap Support
*mc1r*: Melanocortin receptor 1
ML: Maximum Likelihood
*nd4*: NADH dehydrogenase subunit 4
