# Supplementary material for: Digging up the roots of an insular hotspot of genetic diversity: decoupled mito-nuclear histories in the evolution of the Corsican-Sardinian endemic lizard Podarcis tiliguerta
Source: BMC Evol Biol. 2017 Mar 2;17:63. doi: 10.1186/s12862-017-0899-x (PMC5335832; doi:10.1186/s12862-017-0899-x)
Supplement: Additional file 2: Table S2. — Summary statistics of molecular diversity and neutrality tests for each locus and each mitochondrial clade within Podarcis tiliguerta. N: number of sampled gene copies; ns: number of sites of the alignment used for the calculations; S: number of segregating sites; h: number of haplotypes; Hd: haplotype diversity; π: nucleotide diversity; K: average number of pairwise differences; D: Tajima’s D values (1989). Significance values for the Tajima’s D tests based on 1000 coalescent simulations are shown next to the values (ns: P ≥ 0.05; *: 0.01 ≤ P < 0.05; **: 0.001 ≤ P < 0.01; ***: P < 0.001). (PDF 158 kb) [file 12862_2017_899_MOESM2_ESM.pdf]

Additional Table S2. **Summary statistics of molecular diversity and neutrality tests for each locus and each mitochondrial clade within *Podarcis tiliguerta*.** L1-L4: mitochondrial Lineage 1- 4; N: number of sampled gene copies; ns: number of sites of the alignment used for the calculations; S: number of segregating sites; h: number of haplotypes; Hd: haplotype diversity; π: nucleotide diversity; K: average number of pairwise differences; *D*: Tajima's *D* values (1989). Significance values for the Tajima's *D* tests based on 1000 coalescent simulations are shown next to the values (ns: $P \geq 0.05$; *: $0.01 \leq P < 0.05$; **: $0.001 \leq P < 0.01$; ***: $P < 0.001$).

Locus	Clade	n	Sites	S	h	Hd	π	K	*D*	
acm4	*P. tiliguerta*	216	401	60	88	0.913	0.006646	2.6187	-2.32575	**
	Corsica	66	401	29	39	0.959	0.009761	3.9044	-1.28656	ns
	Sardinia	150	401	50	62	0.893	0.00558	2.2041	-2.35256	**
	L1	50	401	23	30	0.943	0.009173	3.6694	-1.02618	ns
	L2	16	401	14	13	0.967	0.010515	4.2167	-0.26328	ns
	L3	72	401	35	37	0.924	0.006694	2.651	-2.06972	*
	L4	78	401	35	38	0.925	0.00555	2.2035	-2.22588	**
mc1r	*P. tiliguerta*	250	614	83	103	0.921	0.004168	2.5053	-2.53499	***
	Corsica	92	614	45	42	0.905	0.00378	2.2984	-2.38731	**
	Sardinia	158	614	65	78	0.95	0.004864	2.9429	-2.3681	**
	L1	70	614	40	37	0.932	0.004094	2.4894	-2.33519	**
	L2	22	614	17	15	0.957	0.004308	2.645	-1.58268	ns
	L3	80	614	47	45	0.922	0.00435	2.6402	-2.34076	**
	L4	78	614	45	50	0.981	0.006179	3.7632	-2.08107	*
12S	*P. tiliguerta*	171	371	40	40	0.955	0.037244	11.5827	0.90019	ns
	Corsica	81	371	22	18	0.898	0.018497	5.9929	0.73042	ns
	Sardinia	90	371	31	27	0.929	0.025723	8.5658	0.4508	ns
	L1	66	371	15	12	0.856	0.010855	3.5277	0.35015	ns
	L2	15	371	11	7	0.819	0.011863	4.1048	0.43727	ns
	L3	43	371	17	14	0.836	0.007825	2.6213	-1.19325	ns
	L4	47	371	20	14	0.883	0.013044	4.4218	-0.36561	ns
nd4	*P. tiliguerta*	149	862	245	105	0.994	0.087405	71.4973	1.03107	ns
	Corsica	81	862	182	59	0.989	0.054942	46.6454	0.44461	ns
	Sardinia	68	862	173	47	0.987	0.067794	55.7941	1.46715	ns
	L1	66	862	116	48	0.986	0.027385	23.2769	-0.23876	ns
	L2	15	862	61	11	0.933	0.026633	22.7714	0.68082	ns
	L3	22	862	40	16	0.97	0.010289	8.4675	-0.89154	ns
	L4	46	862	132	31	0.978	0.041575	35.5469	0.5693	ns
