# Supplementary material for: Digging up the roots of an insular hotspot of genetic diversity: decoupled mito-nuclear histories in the evolution of the Corsican-Sardinian endemic lizard Podarcis tiliguerta
Source: BMC Evol Biol. 2017 Mar 2;17:63. doi: 10.1186/s12862-017-0899-x (PMC5335832; doi:10.1186/s12862-017-0899-x)

Additional Figure S1. Results of statistical parsimony network analyses based on mtDNA data: Sub-networks recovered under the 95% limit for a parsimony connection. Haplotypes are represented by circles with size proportional to their frequencies, coloured according to the main mtDNA lineages, and labelled according to the sublineages recovered in the phylogenetic analysis (see text for more details). Small white circles represent unsampled or extinct haplotypes.

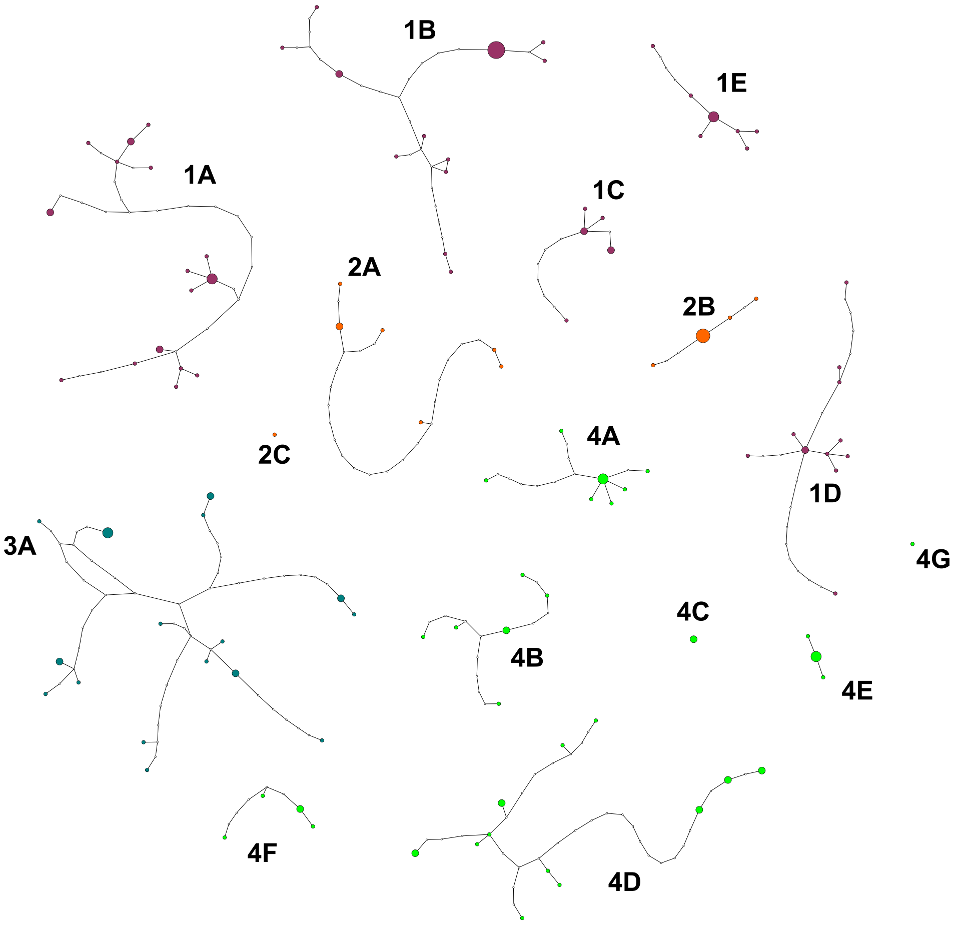

Supplement: Additional file 3: Figure S1. — Results of statistical parsimony network analyses based on mtDNA data. Sub-networks recovered under the 95% limit for a parsimony connection. Haplotypes are represented by circles with size proportional to their frequencies, coloured according to the main mtDNA lineages and labelled according to the sublineages recovered in the phylogenetic analysis (see text for more details). Small white circles represent ‘unsampled or extinct haplotypes. (PDF 86 kb) [file 12862_2017_899_MOESM3_ESM.pdf]
