# Supplementary material for: Digging up the roots of an insular hotspot of genetic diversity: decoupled mito-nuclear histories in the evolution of the Corsican-Sardinian endemic lizard Podarcis tiliguerta
Source: BMC Evol Biol. 2017 Mar 2;17:63. doi: 10.1186/s12862-017-0899-x (PMC5335832; doi:10.1186/s12862-017-0899-x)

Additional Figure S2. Bayesian phylogenetic tree of *Podarcis* based on mitochondrial sequences (*12S* and *nd4*). Bayesian Posterior Probabilities values > 0.90 are reported above the nodes; bootstrap values of the Maximum Likelihood analysis >50 are reported below the nodes.

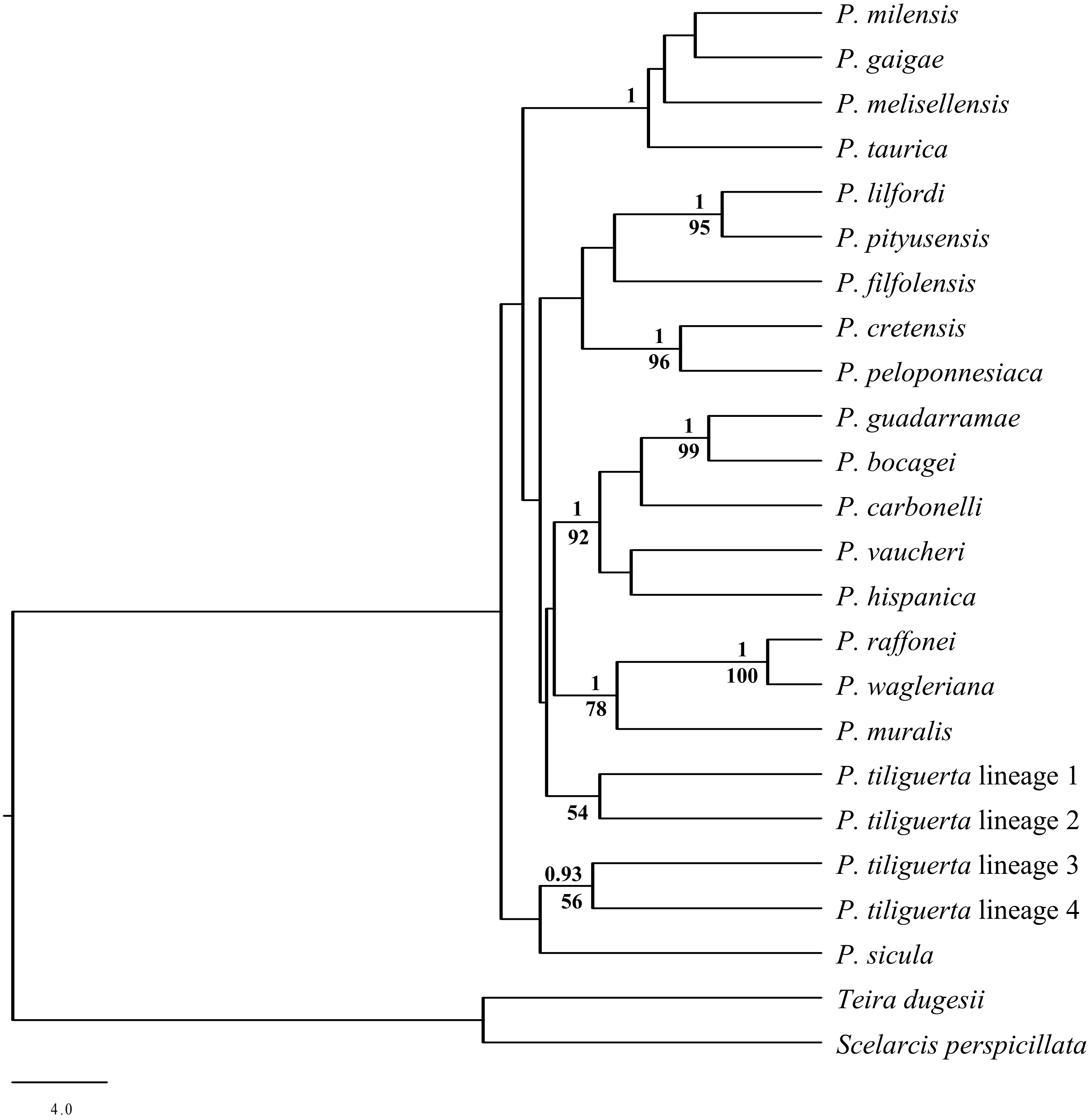

Supplement: Additional file 4: Figure S2. — Bayesian phylogenetic tree of Podarcis based on mitochondrial sequences (12S and nd4). Bayesian Posterior Probabilities values > 0.90 are reported above the nodes; bootstrap values of the Maximum Likelihood analysis > 50 are reported below the nodes. (PDF 72 kb) [file 12862_2017_899_MOESM4_ESM.pdf]
