# Supplementary material for: Digging up the roots of an insular hotspot of genetic diversity: decoupled mito-nuclear histories in the evolution of the Corsican-Sardinian endemic lizard Podarcis tiliguerta
Source: BMC Evol Biol. 2017 Mar 2;17:63. doi: 10.1186/s12862-017-0899-x (PMC5335832; doi:10.1186/s12862-017-0899-x)

Additional Figure S3. Statistical parsimony network showing the genealogical relationships between the *mc1r* haplotypes inferred for nine *Podarcis* species based on 946 sequences retrieved from GenBank [57-60, 80], generated in this study (*P. tiliguerta*), and unpublished (*P. wagleriana*; Salvi et al. in prep). Haplotypes are represented with circles with size proportional to their frequency; small white circle represent 'missing' or extinct haplotypes.

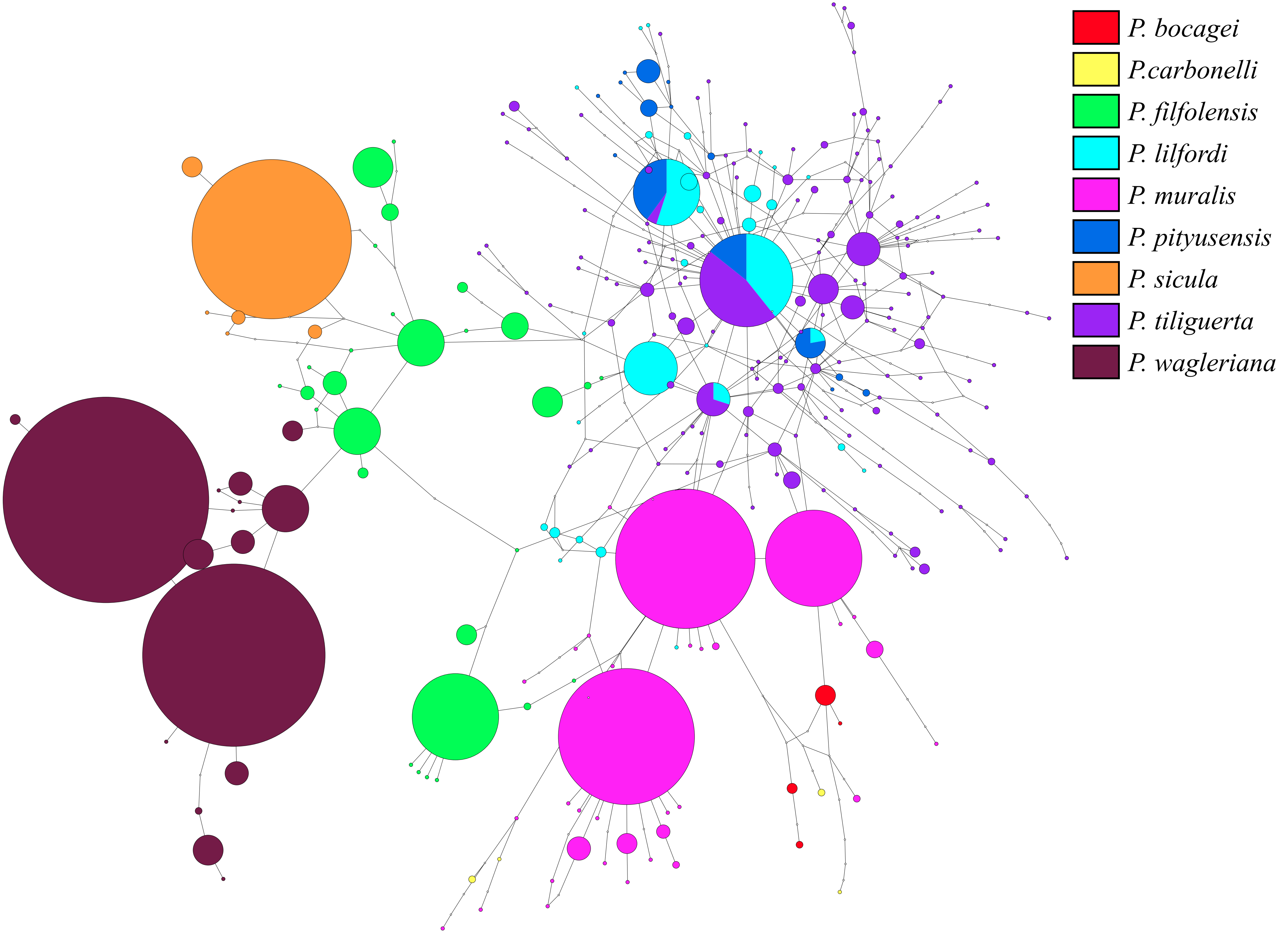

Supplement: Additional file 5: Figure S3. — Statistical parsimony network showing the genealogical relationships between the mc1r haplotypes inferred for nine Podarcis species based on 946 sequences retrieved from Genbank [57–60, 80], generated in this study (P. tiliguerta) and unpublished (P. wagleriana; Salvi et al. in prep.). Haplotypes are represented by circles with size proportional to their frequency; small white circles represent ‘missing’ or extinct haplotypes. (PDF 104 kb) [file 12862_2017_899_MOESM5_ESM.pdf]
