# Supplementary material for: Digging up the roots of an insular hotspot of genetic diversity: decoupled mito-nuclear histories in the evolution of the Corsican-Sardinian endemic lizard Podarcis tiliguerta
Source: BMC Evol Biol. 2017 Mar 2;17:63. doi: 10.1186/s12862-017-0899-x (PMC5335832; doi:10.1186/s12862-017-0899-x)
Supplement: Additional file 6: Table S3. — Result of analyses of molecular variance (AMOVA) based on partitions defined by mtDNA variation of Podarcis tiliguerta. ns: P ≥ 0.05; *: 0.01 ≤ P < 0.05; **: 0.001 ≤ P < 0.01; ***: P < 0.001. (PDF 96 kb) [file 12862_2017_899_MOESM6_ESM.pdf]

Additional Table S3. Result of analyses of molecular variance (AMOVA) based on partitions defined by mtDNA variation of *Podarcis tiliguerta*. ns: P≥0.05; *: 0.01 ≤ P < 0.05; **: 0.001 ≤ P < 0.01; ***: P < 0.001.

Structure			Locus	Percentage of variation			Fixation indices					
**Data set**	**Groups**	**Populations**		**Among groups**	**Among populations within groups**	**Within populations**	F_{SC}		F_{ST}		F_{CT}	
P. tiliguerta	Islands	Lineages										
			acm4	0.81	4.09	95.1	0.0412	***	0.049	***	0.0081	ns
			mc1r	2.32	2.93	94.75	0.03	***	0.0525	***	0.0232	ns
			mtDNA	3.19	73.9	22.92	0.7633	***	0.7708	***	0.0319	ns
P. tiliguerta	-	Islands										
			acm4		3.09	96.91			0.0309	***		
			mc1r		3.97	96.03			0.0397	***		
			mtDNA		49.61	50.39			0.4961	***		
P. tiliguerta	-	Lineages										
			acm4		4.81	95.19			0.0481	***		
			mc1r		4.48	95.52			0.0448	***		
			mtDNA		73.89	23.11			0.7689	***		
Corsica	-	Lineages										
			acm4		7.09	92.91			0.0709	**		
			mc1r		3.28	96.72			0.0328	*		
			mtDNA		77.93	22.07			0.7793	***		
Sardinia	-	Lineages										
			acm4		3.13	96.87			0.0313	***		
			mc1r		2.92	97.08			0.0292	***		
			mtDNA		74.58	25.42			0.7458	***		
