# Supplementary material for: Digging up the roots of an insular hotspot of genetic diversity: decoupled mito-nuclear histories in the evolution of the Corsican-Sardinian endemic lizard Podarcis tiliguerta
Source: BMC Evol Biol. 2017 Mar 2;17:63. doi: 10.1186/s12862-017-0899-x (PMC5335832; doi:10.1186/s12862-017-0899-x)

Additional Figure S2. Patterns of correlation between genetic distances and geographic distances at nuclear loci. Scatterplots of the genetic distance (F_{ST} and Phi_{ST}) versus geographic distance (km) among population pairs of *Podarcis tiliguerta* at the loci *mc1r* and *acm4*. The reduced major xis (RMA) regression lines are shown.

acm4

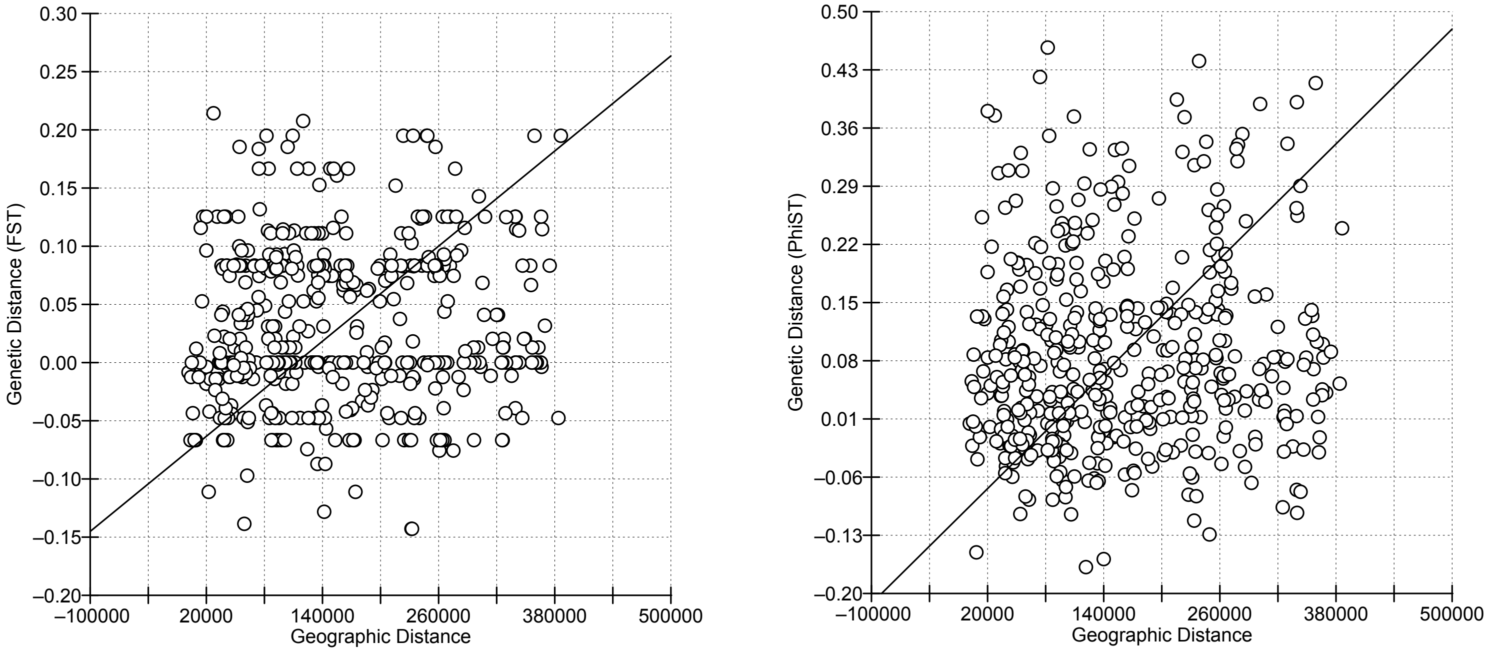

mc1r

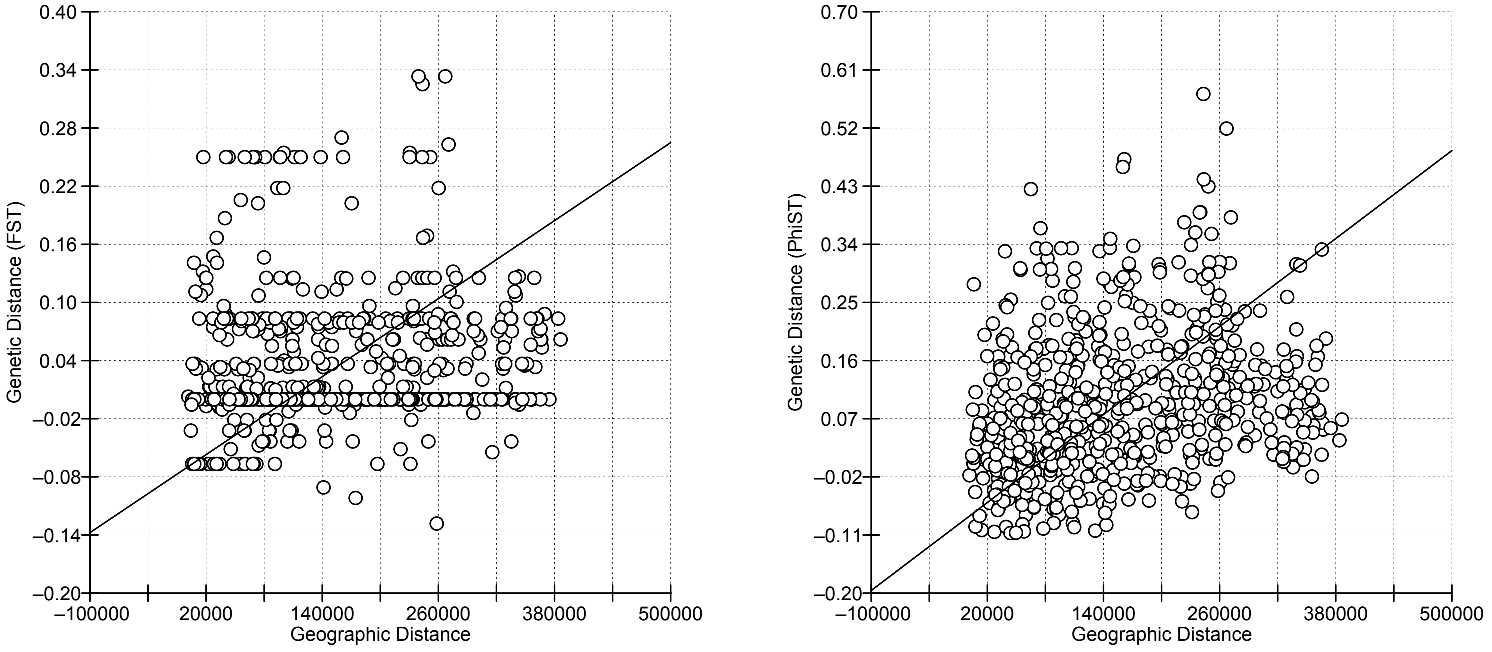

Supplement: Additional file 7: Figure S4. — Patterns of correlation between genetic distances and geographic distance at nuclear loci. Scatterplots of the genetic distances (FST and PhiST) versus geographic distances (km) among population pairs of Podarcis tiliguerta at the loci acm4 and mc1r. The reduced major axis (RMA) regression lines are shown. (PDF 347 kb) [file 12862_2017_899_MOESM7_ESM.pdf]
