# Supplementary material for: Digging up the roots of an insular hotspot of genetic diversity: decoupled mito-nuclear histories in the evolution of the Corsican-Sardinian endemic lizard Podarcis tiliguerta
Source: BMC Evol Biol. 2017 Mar 2;17:63. doi: 10.1186/s12862-017-0899-x (PMC5335832; doi:10.1186/s12862-017-0899-x)
Supplement: Additional file 1: Table S1. — Summary results of haplotype phasing based on data from cloning. For each individual heterozygote sites are highlighted in green and the corresponding sequence of each replicated colony is reported. Nucleotide states in red font included in grey boxes indicate instances of nucleotide misincorporaitons (PCR/replication errors) in colonies and were solved following a parsimony approach. (PDF 103 kb) [file 12862_2017_899_MOESM1_ESM.pdf]

Additional Table S1 – Summary results of haplotype phasing based on data from cloning. For each individual heterozygote sites are highlighted in green and the corresponding sequence of each replicated colony is reported. Nucleotide states in red font included in grey boxes indicate instances of nucleotide misincorporaitons (PCR/replication errors) in colonies and were solved following a parsimony approach.

SAMPLE / LOCUS	8	11	16	34	65	67	68	74	79	90	98	114	131	134	137	143	148	152	158	159	179	197	215	219	228	234	239	249	252	281	284	300	324	344	347	354	355	357	359	367	402	403	412	424	431	438	443	460	461	464	466	473	482	485	489	491	502	507	515	519	527	542	543	544	552	564
master_db1693	Y										Y										Y			C			Y		G			G		Y			G	C					T																	C					T	
db1693_CLONE1	C										T										T			-			T		G			G		C			G	C					T																	C					C	
db1693_CLONE2	C										T										T			C			T		G			G		C			-	C					T																	T					T	
db1693_CLONE3	C										T										T			C			T		G			G		C			G	C					T																	A					T	
db1693_CLONE4	T										C										C			C			C		A			A		T			G	C					T																	C					T	
db1693_CLONE5	T										C										C			C			C		G			G		T			G	C					T																	C					C	
db1693_CLONE6	T										C										C			C			C		G			G		T			G	-					C																	C					T	
db1693_phase_A	C										T										T			C			T		G			G		C			G	C					T																	C					T	
db1693_phase_B	T										C										C			C			C		G			G		T			G	C					T																	C					T	
master_164a			A		Y	T					Y							S																							A						Y						Y					T					-	G		
164a_CLONE1			A		C	T					C							G																							A						C						C					T					-	G		
164a_CLONE2			G		C	T					C							G																							A						C						C					C					-	G		
164a_CLONE3			A		C	T					C							G																							G						T						T					T					-	A		
164a_CLONE4			A		T	T					T							C																							A						T						T					T					-	G		
164a_CLONE5			A		T	C					T							C																							A						T						T					T					C	G		
164a_CLONE6			A		T	C					T							C																							A						T						T					T					C	G		
164a_phase_A			A		C	T					C							G																							A						C						C					T					-	G		
164a_phase_B			A		T	T					T							C																							A						T						T					T					-	G		
master_342a								Y				A																			Y														R									Y												
342a_CLONE1								T				A																			T														A									T												
342a_CLONE2								T				A																			C														G									C												
342a_CLONE3								C				G																			C														G									C												
342a_phase_A								T				A																			T														A									T												
342a_phase_B								C				A																			C														G									C												
master_388a					Y				Y					Y												A		G																							T											Y				Y
388a_CLONE1					T				T					T												G		G																							C											T				C
388a_CLONE2					T				T					T												A		G																							T											T				T
388a_CLONE3					T				T					T												A		G																							T											T				C
388a_CLONE4					T				T					T												A		G																							T											T				C
388a_CLONE5					C				C					C												A		G																							T											C				T
388a_CLONE6					C				C					C												A		A																							T											C				T
388a_phase_A					T				T					T												A		G																							T											T				C
388a_phase_B					C				C					C												A		G																							T											C				T
master_401	C				Y						Y		G			S						S													G							T						T	Y	M							G		T		C					
401_CLONE1	T				T						T		G			C						G													G							T						T	T	A							-		T		T					
401_CLONE2	C				T						T		T			C						G													G							T						T	C	C							G		T		C					
401_CLONE3	C				T						T		G			C						G													A							T						C	C	C							G		T		C					
401_CLONE4	C				T						T		G			C						G													G							C						T	T	A							G		T		C					
401_CLONE5	C				C						C		G			G						C													G							T						T	T	A							G		T		C					
401_CLONE6	C				C						C		G			G						C													G							T						T	T	A							G		C		C					
401_phase_A	C				T						T		G			C						G													G							T						T	C	C							G		T		C					
401_phase_B	C				C						C		G			G						C													G							T						T	T	A							G		T		C					
master_183d	Y										Y						Y						Y	C	R														C	A				A		T																				
183d_CLONE1	T										T						C						C	-	G														C	A				A		T																				
183d_CLONE2	T										T						C						C	C	G														C	A				G		T																				
183d_CLONE3	T										C						T						T	C	A														C	A				A		C																				
183d_CLONE4	C										C						T						T	C	A														T	T				A		T																				
183d_CLONE5	C										C						T						T	C	A														C	A				A		T																				
183d_phase_A	T										T						C						C	C	G														C	A				A		T																				
183d_phase_B	C										C						T						T	C	A														C	A				A		T																				
master_STI															R										R		Y			C											A						Y					G			T					Y						
STI_CLONE1															A										G		T			C											G						C					G			C					C						
STI_CLONE2															A										G		T			C											A						C					G			T					C						
STI_CLONE3															A										G		T			T											A						C					G			T					C						
STI_CLONE4															G										A		C			C											A						T					G			T					T						
STI_CLONE5															G										A		C			C											A						T					A			T					T						
STI_phase_A															A										G		T			C											A						C					G			T					C						
STI_phase_B															G										A		C			C											A						T					G			T					T						
master_LIM1				T	Y		G			C									Y	G													T			G									R											Y										
LIM1_CLONE1				C	T		G			C									C	G													T			G									A											C										
LIM1_CLONE2				T	T		G			C									C	G													T			A									A											C										
LIM1_CLONE3				T	T		G			C									C	G													T			G									G											T										
LIM1_CLONE4				T	T		G			C									C	G													T			G									A											C										
LIM1_CLONE5				T	C		G			C									T	A													C			G									G											T										
LIM1_CLONE6				T	C		A			G									T	G													T			G									G											C										
LIM1_phase_A				T	T		G			C									C	G													T			G									A											C										
LIM1_phase_B				T	C		G			C									T	G													T			G									G											T										
